# Using Negative Control Outcomes and Difference-in-Differences Analysis to Estimate Treatment Effects in an Entirely Treated Cohort: The Effect of Ivacaftor in Cystic Fibrosis

**DOI:** 10.1093/aje/kwab263

**Published:** 2021-11-09

**Authors:** Simon J Newsome, Rhian M Daniel, Siobhán B Carr, Diana Bilton, Ruth H Keogh

**Keywords:** causal inference, cystic fibrosis, cystic fibrosis transmembrane conductance regulator (CFTR) modulators, difference-in-differences analysis, ivacaftor, longitudinal data, negative control outcomes

## Abstract

When an entire cohort of patients receives a treatment, it is difficult to estimate the treatment effect in the treated because there are no directly comparable untreated patients. Attempts can be made to find a suitable control group (e.g., historical controls), but underlying differences between the treated and untreated can result in bias. Here we show how negative control outcomes combined with difference-in-differences analysis can be used to assess bias in treatment effect estimates and obtain unbiased estimates under certain assumptions. Causal diagrams and potential outcomes are used to explain the methods and assumptions. We apply the methods to UK Cystic Fibrosis Registry data to investigate the effect of ivacaftor, introduced in 2012 for a subset of the cystic fibrosis population with a particular genotype, on lung function and annual rate (days/year) of receiving intravenous (IV) antibiotics (i.e., IV days). We consider 2 negative control outcomes: outcomes measured in the pre-ivacaftor period and outcomes among persons ineligible for ivacaftor because of their genotype. Ivacaftor was found to improve lung function in year 1 (an approximately 6.5–percentage-point increase in ppFEV_1_), was associated with reduced lung function decline (an approximately 0.5–percentage-point decrease in annual ppFEV_1_ decline, though confidence intervals included 0), and reduced the annual rate of IV days (approximately 60% over 3 years).

## Abbreviations

CF: cystic fibrosis
CI: confidence interval
CTE: causal treatment effect
DAG: directed acyclic graph
IV: intravenous
NCCTE: negative-control–corrected treatment effect
NCE: negative control effect
NCO: negative control outcome
NTE: naive treatment effect
ppFEV_1_: percent predicted forced expiratory volume in 1 second
SWIG: single-world intervention graph

Randomized controlled trials are the gold standard for estimating treatment effects but are typically infeasible for estimating long-term effects. Observational data provide opportunities to estimate such effects, under strong assumptions. A key assumption is positivity, meaning individuals have a probability less than 1 of receiving (or not) the treatment, given any covariates controlled in the analysis (1).

One situation where this assumption is not met is when an entire cohort of patients receives treatment. Then it is difficult to estimate a treatment effect because we do not observe contemporary untreated individuals. It may be possible to identify a comparable group who could not receive treatment, for example, historical controls prior to its availability. However, resultant analyses make the strong assumption of no differences between the control and treatment group that affect the outcome, except the treatment itself and its consequences. We consider estimation of causal treatment effects (CTEs) in this setting through an investigation of the effect of the disease-modifying treatment ivacaftor in people with cystic fibrosis (CF).

In the United Kingdom, approximately 10,500 persons have CF (2). The most seriously affected organ is the lung, with long-term deterioration in lung function observed. Ivacaftor has been available in the United Kingdom since 2012, and approximately 5% of the UK CF population—those with a particular CF-causing gating mutation—are eligible to receive it.

Randomized controlled trials have found that ivacaftor improves lung function and reduces the incidence of pulmonary exacerbations (3–5). Ramsey et al. (3) reported that in patients aged 12 years or older with the specified gating mutation, the mean percent predicted forced expiratory volume in 1 second (ppFEV_1_) was 10.5 percentage points higher after 48 weeks of ivacaftor treatment versus placebo, and the ivacaftor group was 55% less likely to have a pulmonary exacerbation. Similar results have been reported in younger children (4, 5). These patients will take ivacaftor for many years, and it is hoped that it will change their slope of lung function decline (“slope-change effect”), as well as improve lung function during the initial treatment phase (“step-change effect”).

In most countries with high CF prevalence, almost all eligible patients now receive ivacaftor, meaning that observational data provide no contemporary controls. Four studies using national patient registry data compared ivacaftor users either with people not receiving ivacaftor because they did not have a gating mutation (“genotype comparison”) or people who were eligible for ivacaftor but in the time period prior to its availability (“time-period comparison”) (6–9). The results were similar to randomized controlled trial findings and also suggested longer-term benefits up to 4 years later. However, even after accounting for baseline differences between the treated and untreated, these studies were prone to bias because of people with different CF-causing mutations having different disease trajectories, or because of general improvements over time in the health of the CF population (10). Lung function decline among people with different CF genotypes has previously been found to be similar (11, 12), but even small differences could result in biased findings. The health of people with CF has been improving over time, which could affect time-period comparisons.

In this paper, we use directed acyclic graphs (DAGs) and single-world intervention graphs (SWIGs) (13) to illustrate assumptions made in the choice of control groups for the genotype and time-period comparisons. We describe how negative control outcomes (NCOs) can be used as a tool to detect bias in treatment effect estimates in this setting and used in combination with the difference-in-differences approach to obtain unbiased estimates of the CTE under weaker assumptions. The methods are applied using data from the UK Cystic Fibrosis Registry (14) to obtain more robust estimates of the effect of ivacaftor on lung function and annual rate (days/year) of intravenous (IV) antibiotic use (i.e., IV days).

## DATA

We use UK Cystic Fibrosis Registry data from 2008–2016. The registry has been described elsewhere (14). Briefly, patients undergo an annual assessment, which captures measures of current health status, events that have occurred since the last assessment, and treatments used. Our analyses include 8,444 people: 467 with a gating mutation, and therefore eligible to receive ivacaftor after its introduction, and 7,977 with a nongating mutation (see Web Figure 1, available at https://doi.org/10.1093/aje/kwab263).

We define 2008–2012 as the pre-ivacaftor period and 2013–2016 as the post-ivacaftor period. Outcomes measured in 2012 were made prior to ivacaftor’s becoming available to eligible patients. Key data for this analysis include treatment (ivacaftor), genotype (gating mutation or nongating mutation), calendar period (pre- or post-ivacaftor period), and outcomes (lung function, annual number of IV days). For lung function, the main analyses use ppFEV_1_. We also present results for 2 other measures of lung function (percent predicted forced vital capacity and percent predicted forced midexpiratory flow). Adjusted analyses use several demographic and clinical variables, as measured in 2008 for analyses in the pre-ivacaftor period and in 2012 for the post-ivacaftor period (see “Analysis” section).

We divide individuals into 4 groups defined by genotype and time period (Table 1). Ivacaftor is only available in group B, this being the group with an eligible genotype (*G* = 1) in the post-ivacaftor period (*P* = 1). The other 3 groups do not receive ivacaftor, either because it is not yet available (A) or because they have an ineligible genotype (D) or both (C). Some individuals appear in both groups A and B or both groups C and D.

**Table 1:** Numbers of People and Observations in 4 Groups of Cystic Fibrosis Patients Based on Genotype (Gating Mutation (*G* = 1) or Nongating Mutation (*G* = 0)) and Time Period (Pre-Ivacaftor (2008–2012) (*P* = 0) or Post-Ivacaftor (2013–2016) (*P* = 1)), UK Cystic Fibrosis Registry, 2008–2016^a^

	**No. of** **People**	**No. of Longitudinal** **Observations**	**No. of** **People**	**No. of Longitudinal** **Observations**
**Genotype**	Group A (***P*** = 0, ***G*** = 1)		Group B (***P*** = 1, ***G*** = 1)	
Gating mutation ($G=1$)	437	1,326	397	1,368
	**Group C (*P* = 0, *G* = 0)**		**Group D (*P* = 1, *G* = 0)**	
Nongating mutation ($G=0$)	6,382	19,067	7,378	24,381

^a^ Many individuals contributed to both groups A and B or both groups C and D.

## METHODS

### Causal treatment effect

The CTE of interest is the effect of ivacaftor on an outcome $Y$ in persons who actually receive the treatment ($X=1$). Let ${Y}^{X=0}$ denote the potential value of *Y* had, contrary to fact, a patient not received ivacaftor, and let ${Y}^{X=1}$ denote the potential value of $Y$ had a patient received ivacaftor. The CTE is the average treatment effect in the treated, and for a continuous outcome (ppFEV_1_) this is measured using the mean difference:

(1) \begin{equation*}\mathrm{CTE}=E\!\big({Y}^{X=1}|X=1\big)-E\!\big({Y}^{X=0}\ |X=1\big).\end{equation*}

The treated cohort corresponds to individuals with the eligible genotype in the post-ivacaftor period. Hence, the CTE can be written as

(2) \begin{equation*} \mathrm{CTE}=E\!\big({Y}^{X=1}|P=1,G=1\big)-E\!\big({Y}^{X=0}\ |P=1,G=1\big). \end{equation*}

Since all patients with the eligible genotype did receive ivacaftor in the post-ivacaftor period, ${Y}^{X=1}$ is equal to the observed $Y$; therefore,

(3) \begin{equation*} \mathrm{CTE}=E\!(Y|P=1,G=1)-E\!\big({Y}^{X=0}\ |P=1,G=1\big). \end{equation*}

The first expectation, $E(Y\vert P=1,G=1)$, can be estimated directly as the mean outcome in individuals with $(P=1, G=1)$ (group B). Assumptions are needed, however, to estimate the second expectation, since ${Y}^{X=0}$ is entirely unobserved in group B. In groups A, C, and D, ${Y}^{X=0}=Y$. Hence, we can estimate $E({Y}^{X=0}|P=0,G=1)=E(Y|P=0, G=1)$ using group A, $E({Y}^{X=0}|P=0,G=0)=E(Y\vert P=0, G=0)$ using group C, and $E({Y}^{X=0}|P=1,G=0)=E(Y\vert P=1,G=0)$ using group D. Corresponding methods for the count outcome (IV days), where the CTE is a rate ratio, are described in Web Appendix 1.

### Naive treatment effect

Previous observational studies have compared people receiving ivacaftor to people in the time period prior to its availability (“time-period comparison”) or people not eligible due to their genotype (“genotype comparison”), typically with adjustment for covariates. We define such comparisons as naive treatment effects (NTEs). The (unadjusted) time-period NTE, comparing groups A and B, is defined as

(4) \begin{equation*} {\mathrm{NTE}}_P\kern0.5em =\mathrm{E}\!\left(Y\vert P=1,G=1\right)-\mathrm{E}\!\left(Y\vert P=0,G=1\right). \end{equation*}

Under the genotype comparison, which compares groups B and D, the NTE is

(5) \begin{equation*} {\mathrm{NTE}}_G\kern0.5em =\mathrm{E}\!\left(Y\vert P=1,G=1\right)-\mathrm{E}\!\left(Y\vert P=1,G=0\right). \end{equation*}

**Figure 1:**
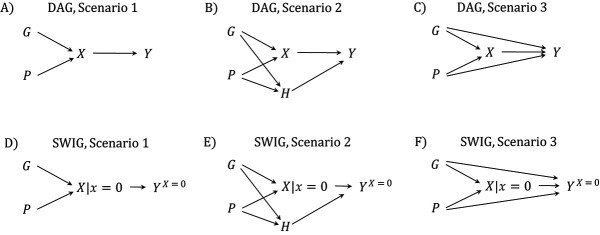
Directed acyclic graphs (DAGs) and single-world intervention graphs (SWIGs) illustrating scenarios 1 (first column), 2 (second column), and 3 (third column). Panels A–C are DAGs showing 3 possible causal pathways between ivacaftor use ($X$), genotype ($G$), time period ($P$), measured covariates of health at baseline ($H$), and the outcome ($Y$). Panels D–F are corresponding SWIGs for the intervention world in which $X$ is set to 0.

Adjusted comparisons are considered below. The above NTEs only correspond to the CTE under the assumption that $E({Y}^{X=0}\ |P=1,G=1)$ is equal to either $\mathrm{E}(Y\vert P=0,G=1)$ or $\mathrm{E}(Y\vert P=1,G=0)$. This assumption is strong, and considerations of its plausibility can be aided by thinking of the 3 scenarios depicted by the DAGs and corresponding SWIGs in Figure 1. We use these causal diagrams to outline the conditions under which the NTEs correspond to the CTE. When they do not, we describe how NCOs can be used in combination with a difference-in-differences analysis to estimate the CTE.

### Scenario 1: *G* and *P* are conditionally independent of *Y* given *X*

In DAG A (Figure 1A), receiving treatment $X$ depends deterministically on $G$ and $P$, but $Y$ is independent of $G$ and $P$ conditional on $X$. From the corresponding SWIG (Figure 1D), we see that ${Y}^{X=0}$ is independent of $G$ and $P$. Thus, $E({Y}^{X=0}|P=1,G=1)=E({Y}^{X=0}|P=p,G=g)$ for any $g,p$. In particular, $E({Y}^{X=0}|P=1,G=1)=E({Y}^{X=0}|P=0,G=1)=E({Y}^{X=0}|P=1,G=0)$. In group A $(P=0,G=1)$ and group D $(P=1,G=0)$, ${Y}^{X=0}=Y$; therefore, $E({Y}^{X=0}|P=1,G=1)=E(Y|P=0,G=1)=E(Y|P=1,G=0)$. It follows that ${\mathrm{NTE}}_P$ and ${\mathrm{NTE}}_G$ correspond to the CTE.

### Scenario 2: *G* and *P* are conditionally independent of *Y* given *X* and *H*

In DAG B (Figure 1B), measured covariates $H$ (baseline health status) are included that affect $Y$ and are also dependent on $G$ and $P$. We see from the corresponding SWIG (Figure 1E) that ${Y}^{X=0}$ is not independent of $G$ and $P$; hence, neither ${\mathrm{NTE}}_P$ nor ${\mathrm{NTE}}_G$ corresponds to the CTE. However, ${Y}^{X=0}$ is conditionally independent of *G* and *P* given *H*, meaning $E({Y}^{X=0}|P=1,G=1,H)=E({Y}^{X=0}|P=p,G=g,H)$ for any $p,g$. Using this, and standardizing to the distribution of $H$ in group B ($P=1,G=1$), the second expectation of the CTE can be written as

(6) \begin{align*} {\displaystyle \begin{array}{l}E\!\left({Y}^{X=0}\ |P=1,G=1\right)\\ {}=\sum \limits_hE\!\left({Y}^{X=0}\ |P=1,G=1,H=h\right)\\\quad\Pr \!\left(H=h|P=1,G=1\right)\\ {}=\sum \limits_hE\!\left({Y}^{X=0}\ |P=p,G=g,H=h\right)\\\quad\Pr\! \left(H=h|P=1,G=1\right).\end{array}} \end{align*}

In all groups except B,

$ {Y}^{X=0}=Y, $

allowing us to write

(7) \begin{align*} {\displaystyle \begin{array}{l}E\!\left({Y}^{X=0}\ |P=1,G=1\right)\\[1ex] {}=\sum \limits_hE\!\left(Y\ |P=0,G=1,H=h\right)\\\quad\Pr\! \left(H=h|P=1,G=1\right)\\[1ex] {}=\sum \limits_hE\!\left(Y\ |P=1,G=0,H=h\right)\\\quad\Pr\! \left(H=h|P=1,G=1\right).\end{array}} \end{align*}

Therefore, the following adjusted NTEs correspond to the CTE under DAG B (Figure 1B):

(8) \begin{align*} &{\mathrm{NTE}}_P^{\mathrm{Adj}} =E\!\left(Y\vert P=1,G=1\right)\nonumber\\&-\sum \limits_hE\!\left(Y\ |P\!=\!0,G\!=\!1,H\!=\!h\right)\Pr \left(H=h|P\!=\!1,G\!=\!1\right)\!. \end{align*}

 

(9) \begin{align*} &{\mathrm{NTE}}_G^{\mathrm{Adj}} =E\!\left(Y\vert P=1,G=1\right)\nonumber\\&-\sum \limits_hE\!\left(Y\ |P\!=\!1,G\!=\!0,H\!=\!h\right)\Pr \left(H=h|P=1,G\!=\!1\right)\!. \end{align*}

These results extend to continuous and multivariable $H$. For these adjusted effects to be estimable requires an assumption that there is overlap in the distribution of $H$ in groups defined by $G$ and $P$. Under the assumption that the effect of $H$ on $Y$ is not modified by $G$ or $P$, the adjusted NTEs can be expressed as conditional differences in expectations (for all $h$):

(10) \begin{align*}& {\mathrm{NTE}}_P^{\mathrm{Con}} =\mathrm{E}\left(Y|\ P=1,G=1,H=h\right)\nonumber\\&-\mathrm{E}\!\left(Y|\ P=0,G=1,H=h\right). \end{align*}

(11) \begin{align*}& {\mathrm{NTE}}_G^{\mathrm{Con}}=\mathrm{E}\left(Y|\ P=1,G=1,H=h\right)\nonumber\\&-\mathrm{E}\!\left(Y|\ P=1,G=0,H=h\right). \end{align*}

### Scenario 3: *G* and *P* are not conditionally independent of *Y*

In DAG C (Figure 1C), there is dependence between $Y$ and $G,P$ conditional on $X$. In an extended version of DAG C in Web Figure 2, we add covariates $H$. The main issue is encompassed in DAG C (Figure 1C), and we focus on this here. The corresponding SWIG (Figure 1F) shows that ${Y}^{X=0}$ is not independent of $G$ and $P$. Now, the unadjusted NTEs (equations 4 and 5) do not correspond to the CTE. If there remains dependence between $Y$ and $G,P$ after conditioning on $H$ as well as $X$, the adjusted NTEs (equations 8 and 9) also do not correspond to the CTE.

NCOs are tools for detecting bias due to unmeasured confounding and other sources in observational studies, and they are defined as outcomes that are not affected by the treatment but have the same associations with other variables as the true outcome of interest (15). The difference-in-differences approach can also be used to estimate treatment effects in the presence of unobserved confounding. Sofer et al. (16) showed the link between NCOs and difference-in-differences analysis, using pretreatment outcome as the NCO. Here we use these tools to detect bias in the NTEs and estimate the CTE under certain assumptions. While these tools have previously been discussed primarily in the context of addressing unmeasured confounding, we use them instead to address bias due to dependence between ${Y}^{X=0}$ and $G,P$. By noting that ${\mathrm{NTE}}_G$ can equivalently be written as ${\mathrm{NTE}}_G=\mathrm{E}(Y\vert P=1,X=1)-\mathrm{E}(Y\vert P=1,X=0)$, it is clear that $G$ is uncontrolled in this difference, and similarly that $P$ is uncontrolled in ${\mathrm{NTE}}_P$. This can be considered a form of unmeasured (or uncontrolled) confounding, as there are backdoor paths from $X$ to $Y$ through $G$ and $P$ that cannot be blocked using a standard analysis because of lack of positivity. The uncontrolled confounder $G$ or $P$ takes the role of an unmeasured confounder ($U$ in Sofer et al. (16)). We consider 2 NCOs that detect the bias in ${\mathrm{NTE}}_G$ and ${\mathrm{NTE}}_P$ due to uncontrolled confounding.

We begin by considering the outcome observed in period $P=0$ (groups A and C) as the NCO. Any difference between $E(Y|P=0,G=1)$ (equivalently $E(Y|P=0,X=1)$) and $E(Y|P=0,G=0)$ (equivalently $E(Y|P=0,X=0)$) cannot be due to treatment, because the outcome measure preceded the treatment. We define the “genotype negative control effect (NCE)” as

(12) \begin{equation*} {\mathrm{NCE}}_G=E\!\left(Y|P=0,G=1\right)-E\!\left(Y|P=0,G=0\right). \end{equation*}

A nonzero ${\mathrm{NCE}}_G$ would indicate that the estimate of ${\mathrm{NTE}}_G$ is due not only to treatment but also to dependence between ${Y}^{X=0}$ and $G$. As in the description of Lipsitch et al. (15), this NCE uses the treatment $X$ and assesses its association with the NCO.

Under certain assumptions, the CTE can be written in terms of the NTE and NCE. The CTE can be written as the difference in differences:

(13) \begin{align*} {\displaystyle \begin{array}{c}\mathrm{CTE}=E\!\left({Y}^{X=1}|P=1,G=1\right)-E\!\left({Y}^{X=0}\ |P=1,G=1\right)\\[1ex] {}=\left\{E\!\left({Y}^{X=1}|P=1,G=1\right)-E\!\left({Y}^{X=0}\ |P=1,G=0\right)\right\}\\[1ex] {}-\left\{E\!\left({Y}^{X=0}\ |P=1,G=1\right)-E\!\left({Y}^{X=0}\ |P=1,G=0\right)\right\}.\end{array}} \end{align*}

The first difference can be written $E({Y}^{X=1}|P=1,G=1)-E({Y}^{X=0}\ |P=1,G=0)=E(Y|P=1,G=1)-E(Y|P=1,G=0)$, which is ${\mathrm{NTE}}_G$. The second difference identifies bias in ${\mathrm{NTE}}_G$. $E({Y}^{X=0}|P=1,G=1)$ cannot be estimated from the data because of lack of positivity. However, we show that the second difference can be estimated under certain assumptions. Consider the model for ${Y}^{X=0}$:

(14) \begin{equation*} E\big({Y}^{X=0}|P=p,G=g\big)=\alpha +{\beta}_Pp+{\beta}_Gg+{\gamma}_{PG} pg. \end{equation*}

Under this model, the second difference in the CTE in equation 13 is $E({Y}^{X=0}|P=1,G=1)-E({Y}^{X=0}\ |P=1,G=0)={\beta}_G+{\gamma}_{PG}$. Additionally, $E({Y}^{X=0}|P=0,G=1)-E({Y}^{X=0}\ |P=0,G=0)={\beta}_G$. It follows that under the assumption that ${\gamma}_{PG}=0$ (i.e., if treatment were set to 0, there would be no product term $P\times G$ in the model for ${Y}^{X=0}$), we have

(15) \begin{align*} {\displaystyle \begin{array}{l}E\!\left({Y}^{X=0}|P=1,G=1\right)-E\!\left({Y}^{X=0}\ |P=1,G=0\right)\\[1ex] {}=E\!\left({Y}^{X=0}|P=0,G=1\right)-E\!\left({Y}^{X=0}\ |P=0,G=0\right).\end{array}} \end{align*}

Under this assumption, the second difference in equation 13 is ${\mathrm{NCE}}_G$ and the CTE can be written as ${\mathrm{NTE}}_G-{\mathrm{NCE}}_G$. We call this the negative-control–corrected treatment effect (NCCTE):

(16) \begin{equation*} {\mathrm{NCCTE}}_G={\mathrm{NTE}}_G-{\mathrm{NCE}}_G.\end{equation*}

An alternative NCO is the outcome observed in genotype group $G=0$. This differs from using the outcome in period 0 as an NCO, as it does not involve an outcome that can be observed in all individuals. Instead of using the outcome measured in 1 time period in individuals in both genotype groups, it makes use of outcomes measured in 2 time periods in individuals with $G=0$. Because the treatment is not given in either time period in the $G=0$ group, we do not assess the association between the treatment and the NCO in this case. However, we show that this NCO can be used to obtain an estimator of the CTE under the same assumptions as those used above. We define the “time-period NCE” as the contrast between the expected outcomes in groups C and D:

(17) \begin{equation*} {\mathrm{NCE}}_P=E\!\left(Y|P=1,G=0\right)-E\!\left(Y|P=0,G=0\right). \end{equation*}

A nonzero ${\mathrm{NCE}}_P$ indicates that the estimate of ${\mathrm{NTE}}_P$ is due not only to treatment but also to dependence between ${Y}^{X=0}$ and $P$. The CTE can be expressed in terms of another difference in differences:

(18) \begin{align*} {\displaystyle \begin{array}{l}\mathrm{CTE}=\left\{E\!\left({Y}^{X=1}|P=1,G\!=\!1\right)-E\!\left({Y}^{X=0}\ |P=0,G\!=\!1\right)\right\}\\[1ex] {}-\left\{E\!\left({Y}^{X=0}\ |P=1,G=1\right)-E\!\left({Y}^{X=0}\ |P=0,G=1\right)\right\}.\end{array}} \end{align*}

Assuming ${\gamma}_{PG}=0$ in equation 14, the CTE can be written as ${\mathrm{NTE}}_P-{\mathrm{NCE}}_P$, and we define

(19) \begin{equation*}{\mathrm{NCCTE}}_P={\mathrm{NTE}}_P-{\mathrm{NCE}}_P. \end{equation*}

**Figure 2:**
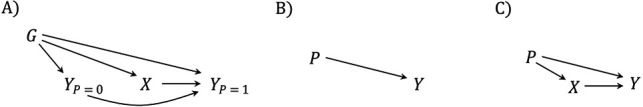
Reformulation of the directed acyclic graph (DAG) in Figure 1C showing the negative control outcomes (NCOs). A) Use of the outcome in period 0 as the NCO. Panels B and C show the DAGs in genotype groups $G=0$ and $G=1$, respectively, when using the outcome in group $G=0$ as the NCO. In panel A, ${Y}_{P=0}$ and ${Y}_{P=1}$ denote the pre- and posttreatment outcomes, respectively, and treatment $X$ occurs after ${Y}_{P=0}.$ In panel A, $G$ is an uncontrolled confounder and is equivalent to the unmeasured confounder $U$ in the work of Sofer et al. (16). In panel C, $P$ is the uncontrolled confounder.

We have shown how a difference-in-differences approach to estimating the CTE corresponds to using an NCO to detect bias in the NTE when there is a violation of the positivity assumption. The NCCTEs correspond to the CTE under weaker assumptions than the NTEs. In our situation there are 2 possible NCOs, corresponding to different difference-in-differences formulae for the CTE (equations 13 and 18). Figure 2 shows reformulations of DAG C (Figure 1C), such that the outcome is shown separately by time period or genotype group. Figure 2A corresponds to the DAG of Sofer et al. (16), with $G$ playing the role of the unmeasured (or uncontrolled) confounder $U$. This illustrates that the outcome in period 0 is not affected by treatment but the 2 outcomes share the same association with $G$. Our second NCO is illustrated in Figures 2B and 2C, where the outcome in group $G=0$ (Figure 2B) is not affected by treatment but the outcomes in the 2 genotype groups share the same association with $P$, which plays the role of the unmeasured confounder in this case. The model for ${Y}^{X=0}$ in equation 14, with ${\gamma}_{PG}=0$, is similar to Sofer et al.’s (16) model for pre- and postexposure outcomes (their equation 3).

We have discussed NCCTEs in the context of DAG C (Figure 1(C)). In practice, it is not known which scenario we are in. NCOs are a way of investigating the validity of the NTE as an estimate of the CTE and, combined with the difference-in-differences analysis, of correcting for bias in the NTE. In scenarios 1 and 2, the NCE is null.

An extended scenario of interest is one in which the effects of $G$ and $P$ on $Y$ are partially mediated through measured covariates $H$ (Web Figure 2). The arguments using NCOs and difference-in-differences analysis can be extended to incorporate adjustment for $H$ (Web Appendix 2). Adjusting for $H$ could result in more efficient estimates of the CTE.

## ANALYSIS

Our aim is to estimate the causal effect of ivacaftor on those eligible to receive it in the post-ivacaftor period using longitudinal data from the UK Cystic Fibrosis Registry. We estimate NTEs, NCEs, and NCCTEs using the time-period and genotype comparisons. We focus here on the continuous outcome ppFEV_1_. For the count outcome, IV days, the treatment effect is quantified by rate ratios after 1, 2, and 3 years of treatment (Web Appendix 1).

The outcome is measured at up to 4 annual review visits ($j=0,1,2,3$) per individual in a given period ($P=0,1$). Let ${Y}_{ij}$ denote the outcome measured for individual $i$ at visit $j$ in a given period. For most individuals in the analysis data set, $j=0$ corresponds to the year 2009 for $P=0$ and 2013 for $P=1$. ${X}_i$ denotes the treatment indicator. The observed treatment status is ${X}_i=1$ for group B and ${X}_i=0$ for groups A, C, and D. We estimate a treatment effect with 2 components, a step-change (St) effect and a slope-change (Sl) effect. The analysis model is

(20) \begin{equation*} E\!\left({Y}_{ij}\ |{X}_i\right)={\beta}_0+{\beta}_{\textrm{St}}\ {X}_i+{\beta}_{\textrm{Sl}}\ {X}_i\ j+{\beta}_J\ j, \end{equation*}

where ${\beta}_{\textrm{St}}=E({Y}_{i0}|{X}_i=1)-E({Y}_{i0}|{X}_i=0)$ represents the step-change effect and ${\beta}_{\textrm{Sl}}=\{E({Y}_{i(j+1)}|{X}_i=1)-E({Y}_{ij}|{X}_i=1)\}-\{E({Y}_{i(j+1)}|{X}_i=0)-E({Y}_{ij}|{X}_i=0)\}$ represents the slope-change effect. Each NTE and NCE comprises a step-change effect and a slope-change effect. ${\mathrm{NTE}}_P$ is estimated by fitting the model in groups A and B ($X$ corresponds to $P$), and ${\mathrm{NTE}}_G$ is estimated by fitting the model in groups D and B ($X$ corresponds to $G$). To estimate the NCEs, the treatment status in one of the groups is switched for the analysis: ${\mathrm{NCE}}_P$ uses groups C and D, setting ${X}_i=1$ for group D (so $X$ corresponds to $P$), and ${\mathrm{NCE}}_G$ uses groups C and A, setting ${X}_{ij}=1$ for group A (so $X$ corresponds to $G$). Each model is fitted using generalized estimating equations, assuming an independence working correlation matrix. Models are refitted with adjustment for variables ${H}_i$ (Table 2) measured in the year prior to visit 0 in each period, giving adjusted NTEs, NCEs, and NCCTEs (see Web Appendix 2).

**Table 2:** Characteristics of Persons in Groups A–D at Baseline, Defined as 2008 for the Pre-Ivacaftor Period and 2012 for the Post-Ivacaftor Period, UK Cystic Fibrosis Registry, 2008–2016

**Variable**	Group A (*P* = 0, *G* = 1) (*n* = 437)			Group B (*P* = 1, *G* = 1) (*n* = 397)			Group C (*P* = 0, *G* = 0) (*n* = 6,382)			Group D (*P* = 1, *G* = 0) (*n* = 7,378)		
	**No.**	**%**	**Mean (SD)**	**No.**	**%**	**Mean (SD)**	**No.**	**%**	**Mean (SD)**	**No.**	**%**	**Mean (SD)**
Ivacaftor use	0	0		397	100		0	0		0	0	
Total no. of postbaseline visits			3.0 (1.1)			3.4 (1.1)			3.0 (1.1)			3.3 (1.0)
Age, years			20.4 (10.8)			22.4 (11.2)			20.9 (11.6)			21.9 (12.6)
Female sex	205	46.9		186	46.9		2,971	46.6		3,465	47.0	
White ethnicity	428	97.9		390	98.2		6,150	96.4		7,043	95.5	
ppFEV_1_			71.0 (23.2)			69.7 (23.2)			71.6 (23.3)			72.0 (23.4)
% predicted forced vital capacity^a^			84.8 (19.4)			84.1 (18.9)			84.0 (19.5)			84.4 (19.6)
% predicted forced midexpiratory flow^a^			56.3 (31.3)			55.9 (32.4)			60.9 (32.8)			58.4 (31.0)
Annual no. of IV antibiotic days			18.4 (28.1)			20.2 (30.5)			17.6 (27.7)			18.6 (28.3)
Infection^b^	358	81.9		350	88.2		4,847	75.9		5,948	80.6	
CF-related diabetes	69	15.8		90	22.7		1,198	18.8		1,751	23.7	
Smoking (yes)	9	2.1		9	2.3		154	2.4		201	2.7	
Mucolytic treatment^c^	223	51.0		264	66.5		2,992	46.9		4,882	65.4	

Abbreviations: CF, cystic fibrosis; IV, intravenous; ppFEV_1_, percent predicted forced expiratory volume in 1 second; SD, standard deviation.

^a^ Percent predicted forced vital capacity was based on 14,556 participants and percent predicted forced midexpiratory flow was based on 5,711 participants, out of a total of 14,594 individuals , many counted more than once, across the 4 groups.

^b^ Baseline infection included infection with *Staphylococcus aureus*, *Pseudomonas aeruginosa*, *Aspergillus fumigatus*, methicillin-resistant *S. aureus* (MRSA), influenza, *Stenotrophomonas maltophilia*, and *Burkholderia cepacia* complex.

^c^ Baseline mucolytic treatment includes acetylcysteine, dornase alfa, hypertonic saline, and mannitol.

Nonparametric bootstrapping with 1,000 resamples was used to obtain 95% confidence intervals (CIs) and *P* values.

## RESULTS

Table 2 summarizes baseline characteristics by group. Comparing groups B and D, a higher proportion of people in the ivacaftor group had an infection (88.2% vs. 80.6%) and White ethnicity (98.2% vs. 95.5%). Comparing groups A and B, the mean age was higher in group B (22.4 years vs. 20.4 years), which also had a slightly higher proportion of infections (88.2% vs. 81.9%), CF-related diabetes (22.7% vs. 15.8%), and use of mucolytic treatment (66.5% vs. 51.0%). Characteristics were otherwise similar across groups. Out of 14,594 people, many counted twice, 7,933 (54.4%) had the maximum of 4 visits postbaseline, with only 1,667 (11.4%) having just 1 visit.

**Figure 3:**
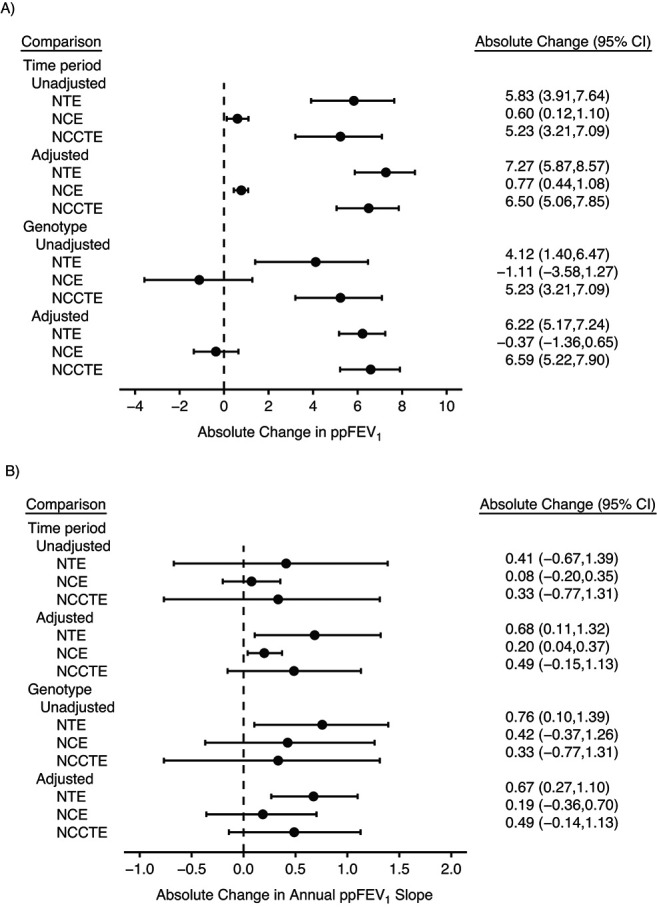
Estimated naive treatment effect (NTE), negative control effect (NCE), and negative-control–corrected treatment effect (NCCTE) of ivacaftor on percent predicted forced expiratory volume in 1 second (ppFEV_1_) among persons with cystic fibrosis, using the time-period comparison (unadjusted: $\mathrm{NT}{\mathrm{E}}_P$, $\mathrm{NC}{\mathrm{E}}_P$, and $\mathrm{NCCT}{\mathrm{E}}_P$; adjusted: $\mathrm{NT}{\mathrm{E}}_P^{\mathrm{Con}}$, $\mathrm{NC}{\mathrm{E}}_P^{\mathrm{Con}}$, and $\mathrm{NCCT}{\mathrm{E}}_P^{\mathrm{Con}}$) and the genotype comparison (unadjusted: $\mathrm{NT}{\mathrm{E}}_G$, $\mathrm{NC}{\mathrm{E}}_G$, and $\mathrm{NCCT}{\mathrm{E}}_G$; adjusted: $\mathrm{NT}{\mathrm{E}}_G^{\mathrm{Con}}$, $\mathrm{NC}{\mathrm{E}}_G^{\mathrm{Con}}$, and $\mathrm{NCCT}{\mathrm{E}}_G^{\mathrm{Con}}$), UK Cystic Fibrosis Registry, 2008–2016. A) Absolute step change in ppFEV_1_ (${\beta}_{\mathrm{St}}$); B) absolute change in the annual ppFEV_1_ slope (${\beta}_{\mathrm{Sl}}$). In the adjusted analysis, results were adjusted for the baseline variables: sex, age, ethnicity, smoking status, cystic-fibrosis–related diabetes, ppFEV_1_, annual number of days of intravenous (IV) antibiotic use (i.e., IV days) (including an indicator of a nonzero count and a linear term for the nonzero counts), use of mucolytic treatment, and bacterial infection. Bars, 95% confidence intervals (CIs).

Results for ppFEV_1_ are shown in Figure 3 (Web Table 1). We focus on the adjusted analysis. The unadjusted results are qualitatively similar, with wider 95% CIs. First consider the step-change effect. In the time-period comparison, the NTE (${\mathrm{NTE}}_P^{\mathrm{Con}}$) estimates a 7.27–percentage-point absolute increase in ppFEV_1_ (95% CI: 5.87, 8.57) in the ivacaftor group. The corresponding NCE (${\mathrm{NCE}}_P^{\mathrm{Con}}$; see Web Appendix 2) estimates a 0.77–percentage-point increase (95% CI: 0.44, 1.08), indicating a small improvement in mean absolute ppFEV_1_ in the post-ivacaftor period in the $G=0$ group. The resulting ${\mathrm{NCCTE}}_P^{\mathrm{Con}}$ estimates a 6.50–percentage-point increase (95% CI: 5.06, 7.85) in ppFEV_1_. In the genotype comparison, ${\mathrm{NTE}}_G^{\mathrm{Con}}$ is estimated to be 6.22 (95% CI: 5.17, 7.24) and ${\mathrm{NCE}}_G^{\mathrm{Con}}$ is estimated to be −0.37 (95% CI: −1.36, 0.65), resulting in an ${\mathrm{NCCTE}}_G^{\mathrm{Con}}$ estimate of a 6.59–percentage-point increase in ppFEV_1_ (95% CI: 5.22, 7.90). The NCE indicates that in the pre-ivacaftor period, mean ppFEV_1_ was slightly lower in persons with the ivacaftor-eligible genotype than in those ineligible.

Slope-change effect estimates suggest a small improvement in lung function decline due to ivacaftor, but 95% CIs for NCCTE estimates include 0. In the time-period comparison, the ${\mathrm{NTE}}_P^{\mathrm{Con}}$ estimates a 0.68–percentage-point absolute improvement in the annual rate of ppFEV_1_ decline (95% CI: 0.11, 1.32). The corresponding estimated ${\mathrm{NCE}}_P^{\mathrm{Con}}$ is 0.20 (95% CI: 0.04, 0.37), giving an ${\mathrm{NCCTE}}_P^{\mathrm{Con}}$ estimate of 0.49 (95% CI: −0.15, 1.13). In the genotype comparison, ${\mathrm{NTE}}_G^{\mathrm{Con}}$ is estimated to be 0.67 (95% CI: 0.27, 1.10) and ${\mathrm{NCE}}_G^{\mathrm{Con}}$ is estimated to be 0.19 (95% CI: −0.36, 0.70), giving an ${\mathrm{NCCTE}}_G^{\mathrm{Con}}$ estimate of a 0.49–percentage-point improvement (95% CI: −0.14, 1.13).

**Figure 4:**
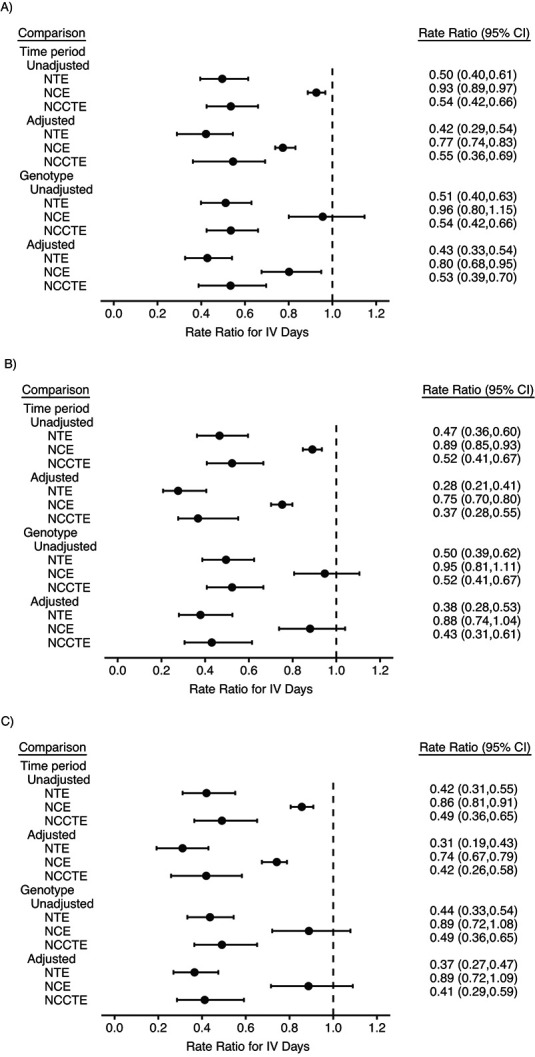
Estimated naive treatment effect (NTE), negative control effect (NCE), and negative-control–corrected treatment effect (NCCTE) of ivacaftor on annual number of days of intravenous (IV) antibiotic use (i.e., IV days) among persons with cystic fibrosis, using the time-period comparison (unadjusted: $\mathrm{NT}{\mathrm{E}}_P$, $\mathrm{NC}{\mathrm{E}}_P$, and $\mathrm{NCCT}{\mathrm{E}}_P$; adjusted: $\mathrm{NT}{\mathrm{E}}_P^{\mathrm{Con}}$, $\mathrm{NC}{\mathrm{E}}_P^{\mathrm{Con}}$, and $\mathrm{NCCT}{\mathrm{E}}_P^{\mathrm{Con}}$) and the genotype comparison (unadjusted: $\mathrm{NT}{\mathrm{E}}_G$, $\mathrm{NC}{\mathrm{E}}_G$, and $\mathrm{NCCT}{\mathrm{E}}_G$; adjusted: $\mathrm{NT}{\mathrm{E}}_G^{\mathrm{Con}}$, $\mathrm{NC}{\mathrm{E}}_G^{\mathrm{Con}}$, and $\mathrm{NCCT}{\mathrm{E}}_G^{\mathrm{Con}}$), UK Cystic Fibrosis Registry, 2008–2016. A) Year 1 (exp(${\gamma}_{X1}))$; B) year 2 (exp(${\gamma}_{X2}))$; C) year 3 (exp(${\gamma}_{X3}))$. The adjusted analysis adjusted for the following baseline variables: sex, age, ethnicity, smoking status, cystic-fibrosis–related diabetes, percent predicted forced expiratory volume in 1 second, IV days (including an indicator of a nonzero count and a linear term for the nonzero counts), use of mucolytic treatment, and bacterial infection. Bars, 95% confidence intervals (CIs).

Results for IV days are shown in Figure 4 (Web Table 2). We focus on the adjusted estimates. In the time-period comparison, according to the ${\mathrm{NTE}}_P^{\mathrm{Con}}$ the rate of IV days was estimated to decrease by 58% (95% CI: 46, 71), 72% (95% CI: 59, 79), and 69% (95% CI: 57, 81) after 1, 2, and 3 years of treatment, respectively. Corresponding estimates of ${\mathrm{NCE}}_P^{\mathrm{Con}}$ were 23% (95% CI: 17, 26), 25% (95% CI: 20, 30), and 26% (95% CI: 21, 33), indicating reductions in IV days in the post-ivacaftor period in the $G=0$ group. The resulting ${\mathrm{NCCTE}}_P^{\mathrm{Con}}$ estimates of the percentage reduction in the rate of IV days were 45% (95% CI: 31, 64), 63% (95% CI: 45, 72), and 58% (95% CI: 42, 74) after 1, 2, and 3 years of treatment, respectively. Results from the genotype comparison were similar.

Results for 2 other measures of lung function (percent predicted forced vital capacity, percent predicted forced midexpiratory flow) (Web Tables 3 and 4) were similar to those for ppFEV_1_. We conducted sensitivity analyses restricting the $G=0$ genotype group to patients who were either heterozygous or homozygous for the phenylalanine 508 deletion (F508del) (Web Tables 5–10) or only those patients who were homozygous for F508del (Web Tables 11–16), corresponding to 90% and 54% of the original $G=0$ group, respectively. The results showed no substantial differences.

## DISCUSSION

We have shown how NCOs can be used to assess whether a control group is suitable for estimating the treatment effect in a group where everyone receives treatment, and how they can be used in combination with the difference-in-differences approach to provide a more robust treatment effect estimate. Previous descriptions of NCOs have focused on unmeasured confounding bias (15–19). Potential bias in our situation is due to an inability to block all paths from $X$ to $Y$ using a standard analysis, which could be considered a form of unmeasured confounding. A key assumption of our methods is that there is no genotype × period product term in the model for the counterfactual outcome under no treatment (equation 14). This is a strong assumption that is not verifiable using the data, though it is weaker than the assumptions made when using NTEs. NCCTEs also provide unbiased treatment effect estimates in further scenarios—for example, allowing for unmeasured variables $U$ affecting $H$ and $Y$ (Web Figure 3). It is of interest to investigate how the assumption made in our difference-in-differences analyses using NCOs could be relaxed. One approach could be the use of synthetic control methods, which make use of pre- and postintervention observations in the group receiving the intervention, and observations in multiple time periods for groups that have not received the intervention (20, 21).

Our NTE estimates for the effect of ivacaftor are similar to results from previous studies, which found that ivacaftor results in a step-change absolute improvement in ppFEV_1_ of 3.2–8.2 percentage points and a decrease in the rate of annual ppFEV_1_ decline of approximately 0.8 percentage points (6, 9). However, these are only unbiased estimates of the treatment effect if the assumptions that $G$ and $P$ are conditionally independent of $Y$ given $X$ (or $X$ and $H$) are valid.

The time-period comparison NCE showed a 0.77–percentage-point absolute increase in ppFEV_1_, indicating a small, non–clinically significant improvement in population average lung function since the introduction of ivacaftor. This means that the NTE slightly overestimates the ivacaftor effect. In the genotype comparison, the NCE was negative, indicating slightly lower ppFEV_1_ in the eligible genotype group versus the ineligible group in the pre-ivacaftor period, but the NCE was also small, as it was in the time-period comparison. This resulted in NCCTE estimates of step-change improvement in ppFEV_1_ of 6.50% and 6.59%, which are similar to the NTE estimates (7.27% to 6.22%) but have wider CIs, correctly reflecting uncertainty in the comparability of the groups.

When considering ivacaftor’s effect on the slope change of lung function, the NCE suggested that some of the NTE estimate was due not to ivacaftor but to general improvements in lung function decline over time. The NCCTE suggests a beneficial effect of ivacaftor, with an estimated absolute improvement in annual rate of decline of 0.49%, though with 95% CIs including 0.

Findings for the ivacaftor effect on rate of IV days were similar to those from previous studies, indicating a treatment benefit that persists at least up to 3 years. The NCE results estimated that in the absence of treatment, the rate of IV days was slightly lower in the $G=1$ group versus the $G=0$ group and in the later time period. NCCTE estimates were therefore slightly attenuated in comparison with the NTEs. Our results support evidence of a long-term clinical benefit of ivacaftor.

## Supplementary Material

Web_Material_kwab263

## References

[1] Hernán MA, Robins JM. Causal Inference: What If. Boca Raton, FL: Chapman & Hall/CRC Press; 2020.

[2] Cystic Fibrosis Trust . UK Cystic Fibrosis Registry Annual Data Report 2019. London, United Kingdom: Cystic Fibrosis Trust; 2020. https://www.cysticfibrosis.org.uk/sites/default/files/2020-12/2019%20Registry%20Annual%20Data%20report_Sep%202020.pdf. Accessed September 20, 2021.

[3] Ramsey BW, Davies J, McElvaney NG, et al. A CFTR potentiator in patients with cystic fibrosis and the *G551D* mutation. *N Engl J Med*. 2011;365(18):1663–1672. 22047557 10.1056/NEJMoa1105185 PMC3230303

[4] Davies JC, Wainwright CE, Canny GJ, et al. Efficacy and safety of ivacaftor in patients aged 6 to 11 years with cystic fibrosis with a *G551D* mutation. *Am J Respir Crit Care Med*. 2013;187(11):1219–1225. 23590265 10.1164/rccm.201301-0153OC PMC3734608

[5] Flume P, Wainwright C, Tullis E, et al. Pulmonary exacerbations in CF patients with the *G551D-CFTR* mutation treated with ivacaftor [abstract]. *J Cyst Fibros*. 2013;12(suppl 1):S63. 10.1016/j.jcf.2017.06.002 28651844

[6] Sawicki GS, McKone EF, Pasta DJ, et al. Sustained benefit from ivacaftor demonstrated by combining clinical trial and cystic fibrosis patient registry data. *Am J Respir Crit Care Med*. 2015;192(7):836–842. 26132840 10.1164/rccm.201503-0578OC

[7] Bessonova L, Volkova N, Higgins M, et al. Data from the US and UK cystic fibrosis registries support disease modification by CFTR modulation with ivacaftor. *Thorax*. 2018;73(8):731–740. 29748252 10.1136/thoraxjnl-2017-210394 PMC6204955

[8] Hubert D, Dehillotte C, Munck A, et al. Retrospective observational study of French patients with cystic fibrosis and a Gly551Asp-*CFTR* mutation after 1 and 2 years of treatment with ivacaftor in a real-world setting. *J Cyst Fibros*. 2018;17(1):89–95. 28711222 10.1016/j.jcf.2017.07.001

[9] Volkova N, Moy K, Evans J, et al. Disease progression in patients with cystic fibrosis treated with ivacaftor: data from national US and UK registries. *J Cyst Fibros*. 2020;19(1):68–79. 31196670 10.1016/j.jcf.2019.05.015

[10] Stevens DP, Marshall BC. A decade of healthcare improvement in cystic fibrosis: lessons for other chronic diseases. *BMJ Qual Saf*. 2014;23(suppl 1):i1–i2. 10.1136/bmjqs-2014-002871 24608544

[11] McKone E, Emerson S, Edwards K, et al. Effect of genotype on phenotype and mortality in cystic fibrosis: a retrospective cohort study. *Lancet*. 2003;361(9370):1671–1676. 12767731 10.1016/S0140-6736(03)13368-5

[12] Sawicki GS, McKone EF, Millar SJ, et al. Patients with cystic fibrosis and a *G551D* or homozygous *F508del* mutation: similar lung function decline [letter]. *Am J Respir Crit Care Med*. 2017;195(12):1673–1676. 10.1164/rccm.201608-1678LE 28617084

[13] Richardson TS . Single world intervention graphs (SWIGs): a unification of the counterfactual and graphical approaches to causality. Seattle, WA: Center for Statistics and the Social Sciences, University of Washington; 2013. (CSSS Working Paper no. 128). https://csss.uw.edu/research/working-papers/single-world-intervention-graphs-swigs-unification-counterfactual-and. Accessed September 20, 2021.

[14] Taylor-Robinson D, Archangelidi O, Carr S, et al. Data resource profile: the UK Cystic Fibrosis Registry. *Int J Epidemiol*. 2018;47(1):9–10e. 29040601 10.1093/ije/dyx196 PMC5837577

[15] Lipsitch M, Tchetgen Tchetgen E, Cohen T. Negative controls: a tool for detecting confounding and bias in observational studies. *Epidemiology*. 2010;21(3):383–388. 20335814 10.1097/EDE.0b013e3181d61eeb PMC3053408

[16] Sofer T, Richardson D, Colicino E, et al. On negative outcome control of unobserved confounding as a generalization of difference-in-differences. *Stat Sci*. 2016;31(3):348–361. 28239233 10.1214/16-STS558 PMC5322866

[17] Smith GD . Negative control exposures in epidemiologic studies. *Epidemiology*. 2012;23(2):351–352. 10.1097/EDE.0b013e318245912c 22317815

[18] Weisskopf MG, Tchetgen Tchetgen EJ, Raz R. On the use of imperfect negative control exposures in epidemiologic studies. *Epidemiology*. 2016;27(3):365–367. 27035687 10.1097/EDE.0000000000000454 PMC12186581

[19] Shi X, Miao W, Nelson J, et al. Multiply robust causal inference with double negative control adjustment for categorical unmeasured confounding. *J R Stat Soc Series B Stat Methodol*. 2020;82(2):521–540. 33376449 10.1111/rssb.12361 PMC7768794

[20] Abadie A, Diamond A, Hainmueller J. Synthetic control methods for comparative case studies: estimating the effect of California’s tobacco control program. *J Am Stat Assoc*. 2010;105(490):493–505.

[21] O’Neill S, Kreif N, Grieve R, et al. Estimating causal effects: considering three alternatives to difference-in-differences estimation. *Health Serv Outcomes Res Methodol*. 2016;16(1-2):1–21. 27340369 10.1007/s10742-016-0146-8 PMC4869762

