# Supplementary material for: Using Negative Control Outcomes and Difference-in-Differences Analysis to Estimate Treatment Effects in an Entirely Treated Cohort: The Effect of Ivacaftor in Cystic Fibrosis
Source: Am J Epidemiol. 2021 Nov 9;191(3):505–15. doi: 10.1093/aje/kwab263 (PMC8914944; doi:10.1093/aje/kwab263)
Supplement: Web_Material_kwab263 [file web_material_kwab263.pdf]

Web Material

Using Negative Control Outcomes and Difference-in-Differences Analysis to Estimate Treatment Effects in an Entirely Treated Cohort: The Effect of Ivacaftor in Cystic Fibrosis

Simon J. Newsome, Rhian M. Daniel, Siobhán B. Carr, Diana Bilton, and Ruth H. Keogh

Contents

Web Appendices 1 and 2

Web Figures 1–3

Web Tables 1–16

WEB APPENDIX 1

Extensions to the Count Outcome

1.1 Defining the treatment effect

The definitions of the causal treatment effect (CTE), naive treatment effect (NTE), negative control effect (NCE) and negative-control-corrected treatment effect (NCCTE) in the main text focused on a continuous outcome (lung function) and the treatment effect was measured using a mean difference. Here we outline extensions to a count outcome (number of IV days), where the treatment effect is measured using a ratio of expected counts (over a 1 year period), which can also be interpreted as a rate ratio. The CTE is defined as

$$\text{CTE} = \frac{E(Y^{X=1} \mid X = 1)}{E(Y^{X=0} \mid X = 1)} = \frac{E(Y^{X=1} \mid P = 1, G = 1)}{E(Y^{X=0} \mid P = 1, G = 1)} = \frac{E(Y \mid P = 1, G = 1)}{E(Y^{X=0} \mid P = 1, G = 1)} \quad \text{(A1)}$$

The NTEs based on the time-period comparison and the genotype comparison are

$$\text{NTE}_P = \frac{E(Y \mid P = 1, G = 1)}{E(Y \mid P = 0, G = 1)} \quad \text{(A2)}$$

$$\text{NTE}_G = \frac{E(Y \mid P = 1, G = 1)}{E(Y \mid P = 1, G = 0)} \quad \text{(A3)}$$

and these correspond to the CTE under the assumptions illustrated in DAG A (main text Figure 1A). We also define the adjusted NTEs

$$\text{NTE}_P^{\text{Adj}} = \frac{\sum_h E(Y|P = 1, G = 1, H = h) \Pr(H = h|P = 1, G = 1)}{\sum_h E(Y \mid P = 0, G = 1, H = h) \Pr(H = h|P = 1, G = 1)} \quad \text{(A4)}$$

and

$$\text{NTE}_G^{\text{Adj}} = \frac{\sum_h E(Y|P = 1, G = 1, H = h) \Pr(H = h|P = 1, G = 1)}{\sum_h E(Y \mid P = 1, G = 0, H = h) \Pr(H = h|P = 1, G = 1)} \quad \text{(A5)}$$

which correspond to the CTE under the assumptions in DAG B (main text Figure 1B). Like mean differences, ratios of expectations (here interpreted as a rate ratio) are collapsible and therefore, under the assumption that the effect of H on Y is not modified by either G or P the adjusted NTEs can be expressed as the ratios of conditional expectations

$$\text{NTE}_P^{\text{Con}} = \frac{E(Y \mid P = 1, G = 1, H = h)}{E(Y \mid P = 0, G = 1, H = h)} \quad \text{(A6)}$$

$$\text{NTE}_G^{\text{Con}} = \frac{E(Y \mid P = 1, G = 1, H = h)}{E(Y \mid P = 1, G = 0, H = h)} \quad \text{(A7)}$$

1.2 Use of negative control outcomes (NCO) for a count outcome

Here we extend the NCO and difference-in-differences methods outlined for the continuous outcome to the context of the count outcome, for under the assumptions in DAG C (main text Figure 1C). For the count outcome the CTE is defined using a ratio of expectations. The difference-in-differences approach therefore becomes a ratio-of-ratios approach for the count outcome. As for the continuous outcome in the main text, we begin by considering the outcome observed in period $P = 0$ (groups A and C) as the NCO. For the count outcome we define the genotype negative control effect (NCE) as

$$\mathrm{NCE}_G = \frac{E(Y|P=0,G=1)}{E(Y|P=0,G=0)} \tag{A8}$$

An NCE_G not equal to 1 indicates that the estimate of the NTE_G is not only due to treatment but also due to dependence between $Y^{X=0}$ and G. The CTE in (A1) can be written as the ratio-of-ratios:

$$\mathrm{CTE} = \frac{E(Y\,|\,P=1,G=1)}{E(Y^{X=0}\,|P=1,G=0)}\frac{E(Y^{X=0}\,|\,P=1,G=0)}{E(Y^{X=0}\,|P=1,G=1)} \tag{A9}$$

The first ratio can be written $\frac{E(Y\,|\,P=1,G=1)}{E(Y^{X=0}\,|P=1,G=0)} = \frac{E(Y\,|\,P=1,G=1)}{E(Y\,|P=1,G=0)}$, which is NTE_G. The second ratio identifies multiplicative bias in NTE_G. Consider the following model for the counterfactual count outcome under no treatment, $Y^{X=0}$,

$$E(Y^{X=0}|P=p,G=g) = \exp(\alpha + \beta_P p + \beta_G g + \gamma_{PG} pg). \tag{A10}$$

Using this we can see that $\frac{E(Y^{X=0}\,|\,P=1,G=0)}{E(Y^{X=0}\,|P=1,G=1)} = \frac{E(Y^{X=0}\,|\,P=0,G=0)}{E(Y^{X=0}\,|P=0,G=1)}$ under the assumption that $\gamma_{PG} = 0$, where the ratio on the right-hand side is NCE_G^{-1}. It follows that the CTE can be written as $\mathrm{NTE}_G/\mathrm{NCE}_G$, which we denote NCCTE_G. Using the outcome observed in the ineligible genotype group $G=0$ as an alternative NCO it can be shown that under the same assumption the CTE can be written as $\mathrm{NTE}_P/\mathrm{NCE}_P$, where $\mathrm{NCE}_P = \frac{E(Y\,|P=1,G=0)}{E(Y\,|P=0,G=0)}$, which we define as NCCTE_P.
This approach can be extended to incorporate adjustment for baseline covariates H, as outlined in Web Appendix 2 for the continuous outcome.

1.3 Analysis for the count outcome

Using the same notation as for the continuous outcome in the main text, the conditional analysis model for estimating the conditional NTEs and NCEs for the count outcome is a negative binomial model with

$$E(Y_{ij}\,|X_i,u_{ij}) = \exp(\gamma_0 + \sum\nolimits_{k=1}^{3} \gamma_{1k}\mathbb{1}[j=k] + \sum\nolimits_{k=1}^{3} \gamma_{Xk}X_i\mathbb{1}[j=k] + u_{ij}) \tag{A11}$$

where $e^{u_{ij}} = \mathrm{Gamma}\left(\frac{1}{\alpha},\alpha\right)$, with α representing an over-dispersion parameter, and where

$$e^{\gamma_{Xj}} = \frac{E\left(Y_{ij}\,|X_i=1,u_{ij}\right)}{E(Y_{ij}\,|X_i=0,u_{ij})}\ (j=1,2,3) \tag{A12}$$

are the adjusted rate ratios in the year following 1, 2 and 3 complete years of treatment. For the ppFEV1 outcome we used outcomes observed at 4 visits $j = 0,1,2,3$. The IV days outcome is measured retrospectively at each visit, e.g. the IV days recorded at the study visit in 2016 refer to IV days over the preceding year. This is why, for the IV days outcome, the data as available only permitted estimation of rate ratios after up to 3 complete years of treatment. As outlined in the main text for the continuous outcome, the adjusted NTEs and NCEs are estimated by fitting the analysis model in different pairs of the groups A, B, C and D. As for the continuous outcome, we also fitted the analysis model for the count outcome with adjustment for H_i, the set of 'baseline' adjustment variables (main text Table 2), measured in the year prior to visit 0 in each period, giving adjusted NTEs, NCEs and NCCTEs.

WEB APPENDIX 2

Extensions to Include Adjustment for Baseline Variables H

The DAG and corresponding SWIG in Web Figure 2 illustrate an extension of DAG C and SWIG F in the main text (Figures 1C and 1F). In the extended DAG, there is dependence between Y and G, P after conditioning on X and H (representing baseline health status). Here we extend the results given in the main text for Scenario (c) to show how NCOs and difference-in-differences can be used to estimate the CTE.

2.1 Using the outcome measured in period 0 as the NCO

The genotype negative control effect (NCE), adjusted for H and standardised to group B is

$$\text{NCE}_G^{\text{Adj}} = \sum_h [E(Y|P=0, G=1, H=h) - E(Y\,|P=0, G=0, H=h)]\,\Pr(H=h|P=1, G=1). \quad \text{(A13)}$$

A non-zero $\text{NCE}_G^{\text{Adj}}$ would indicate that the estimate of $\text{NTE}_G^{\text{Adj}}$ (equation 9 in the main text) is not only due to treatment, but also to dependence between $Y^{X=0}$ and G conditional on H. The CTE (equations 1-3 in the main text) can be written as the difference-in-differences:

$$\begin{aligned}\text{CTE} &= E(Y|P=1, G=1) - \sum_h E(Y^{X=0}|P=1, G=1, H=h)\Pr(H=h|P=1, G=1)\\ &= \left\{E(Y|P=1, G=1) - \sum_h E(Y^{X=0}\,|P=1, G=0, H=h)\Pr(H=h|P=1, G=1)\right\}\\ &\quad - \left\{\sum_h [E(Y^{X=0}|P=1, G=1, H=h) - E(Y^{X=0}\,|P=1, G=0, H=h)]\Pr(H=h|P=1, G=1)\right\}\end{aligned} \quad \text{(A14)}$$

The first difference in the right-hand-side of (A14) is $\text{NTE}_G^{\text{Adj}}$ The second difference identifies the bias in $\text{NTE}_G^{\text{Adj}}$. Consider the following model for $Y^{X=0}$

$$E(Y^{X=0}|P=p, G=g, H=h) = \alpha + \beta_P p + \beta_G g + \beta_H h + \gamma_{PG} pg + \gamma_{PH} ph + \gamma_{GH} gh. \quad \text{(A15)}$$

Under this model the difference in the second term in the CTE in (A14) is $E(Y^{X=0}|P=1, G=1, H=h) - E(Y^{X=0}\,|P=1, G=0, H=h) = \beta_G + \gamma_{PG} + \gamma_{GH} h$. Under this model we also have $E(Y^{X=0}|P=0, G=1, H=h) - E(Y^{X=0}\,|P=0, G=0, H=h) = \beta_G + \gamma_{GH} h$. It follows that under the assumption that $\gamma_{PG} = 0$, in other words that in the absence of treatment, and conditional on H , there is no product term GP in the model for $Y^{X=0}$, we have

$$\begin{aligned}&E(Y^{X=0}|P=1, G=1, H=h) - E(Y^{X=0}\,|P=1, G=0, H=h)\\ &\quad = E(Y^{X=0}|P=0, G=1, H=h) - E(Y^{X=0}\,|P=0, G=0, H=h),\end{aligned} \quad \text{(A16)}$$

Under this assumption the second term in (A14) is $\text{NCE}_G^{\text{Adj}}$, as given in equation (A13), and the CTE can be written as $\text{NTE}_G^{\text{Adj}} - \text{NCE}_G^{\text{Adj}}$. We refer to this as the adjusted negative control corrected treatment effect (NCCTE):

$$\text{NCCTE}_G^{\text{Adj}} = \text{NTE}_G^{\text{Adj}} - \text{NCE}_G^{\text{Adj}} \quad \text{(A17)}$$

If neither P nor G modify the effect of H on Y, then $\text{NCE}_G^{\text{Adj}}$ can be expressed as a conditional difference in expectations (the same for all values of h),

$$\text{NCE}_G^{\text{Con}} = E(Y|P=0,G=1,H=h) - E(Y|P=0,G=0,H=h) \quad \text{(A18)}$$

and then the adjusted NCCTE can be expressed as the difference in conditional differences, $\text{NCCTE}_G^{\text{Con}} = \text{NTE}_G^{\text{Con}} - \text{NCE}_G^{\text{Con}}$.

2.2 Using the outcome measured in genotype group 0 as the NCO

The time-period negative control effect (NCE), adjusted for H and standardised to group B is

$$\text{NCE}_P^{\text{Adj}} = \sum_h [E(Y|P=1,G=0,H=h) - E(Y\,|P=0,G=0,H=h)]\,\Pr(H=h|P=1,G=1). \quad \text{(A19)}$$

The CTE can be also be written as the difference-in-differences

$$\text{CTE} = \left\{E(Y|P=1,G=1) - \sum_h E(Y\,|P=0,G=1,H=h)\,\Pr(H=h|P=1,G=1)\right\} - \left\{\sum_h [E(Y^{X=0}|P=1,G=1,H=h) - E(Y\,|P=0,G=1,H=h)]\,\Pr(H=h|P=1,G=1)\right\} \quad \text{(A20)}$$

The first difference is equal to $\text{NTE}_P^{\text{Adj}}$ in equation (8) in the main text. Under the assumption that $\gamma_{PG} = 0$ in the model for $Y^{X=0}$ in equation (A15) we have

$$E(Y^{X=0}|P=1,G=1,H=h) - E(Y^{X=0}\,|P=0,G=1,H=h) = E(Y^{X=0}|P=1,G=0,H=h) - E(Y^{X=0}\,|P=0,G=0,H=h). \quad \text{(A21)}$$

Under this assumption the second term in (A20) is $\text{NCE}_P^{\text{Adj}}$, as given in equation (A19), and the CTE can be written as $\text{NTE}_P^{\text{Adj}} - \text{NCE}_P^{\text{Adj}}$, giving the adjusted NCCTE

$$\text{NCCTE}_P^{\text{Adj}} = \text{NTE}_P^{\text{Adj}} - \text{NCE}_P^{\text{Adj}} \quad \text{(A22)}$$

If neither P nor G modify the effect of H on Y, then $\text{NCE}_P^{\text{Adj}}$ can be expressed as a conditional difference in expectations (the same for all values of h),

$$\text{NCE}_P^{\text{Con}} = E(Y|P=1,G=0,H=h) - E(Y|P=0,G=0,H=h) \quad \text{(A23)}$$

and then the adjusted NCCTE can be expressed as the difference in conditional differences, $\text{NCCTE}_P^{\text{Con}} = \text{NTE}_P^{\text{Con}} - \text{NCE}_P^{\text{Con}}$.

Web Figure 1. Flowchart of people included in analysis. People were excluded from our analysis if they were aged under 6 in 2016, had received a transplant before 2008, had no lung function data, or did not have at least two consecutive annual visits. A small number were excluded due to receiving ivacaftor despite not having an eligible genotype. For patients who turned 6 between 2008 and 2016, only data after age 6 were included. Patients who received a transplant or died between 2008 and 2016 were censored at the visit prior to this.

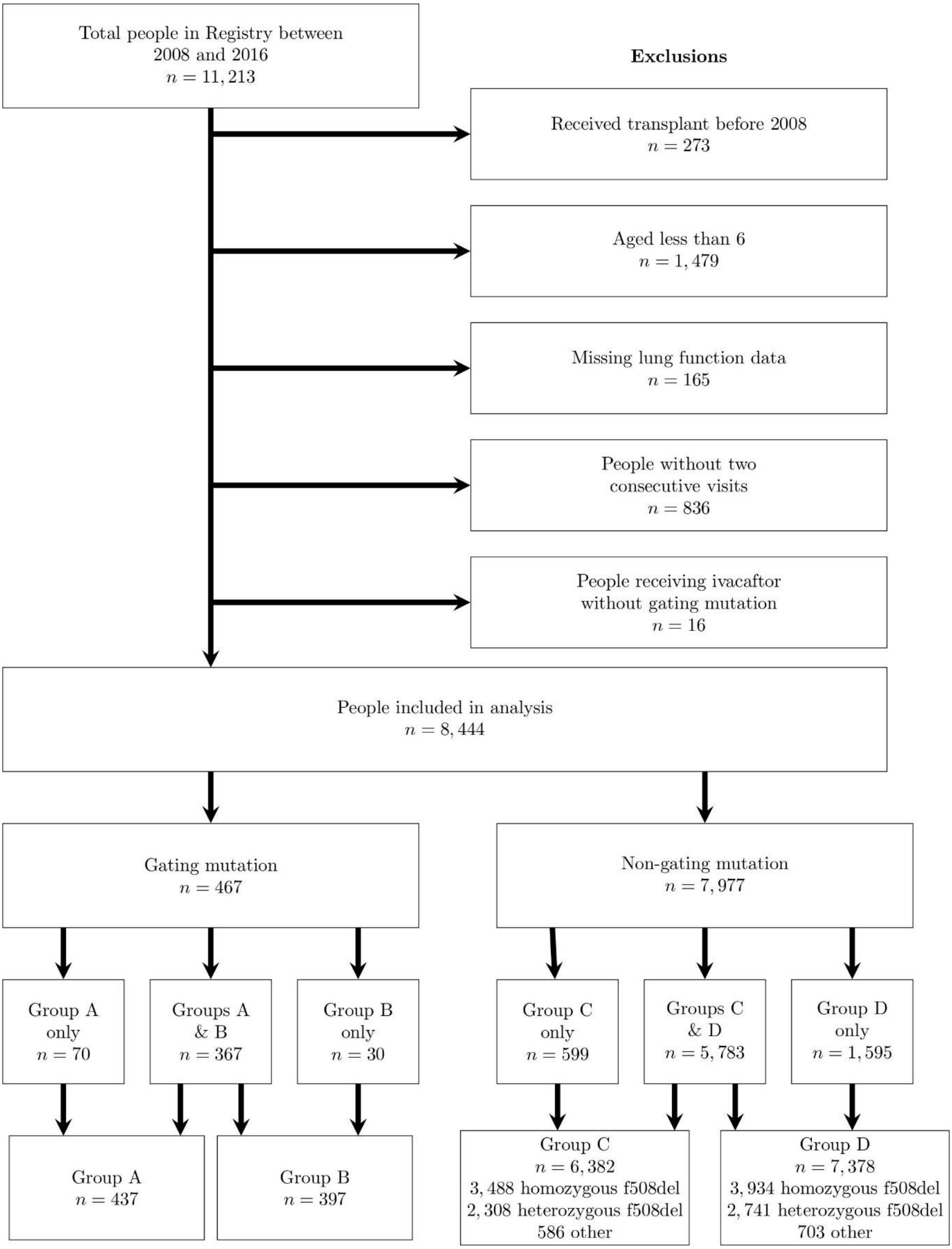

Web Figure 2. Directed acyclic graph (DAG) showing causal pathways between ivacaftor (X), genotype (G), time period (P), measured covariates of health at baseline (H), and outcome (Y) and corresponding single world intervention graph (SWIG) for the intervention world in which X is set to 0.

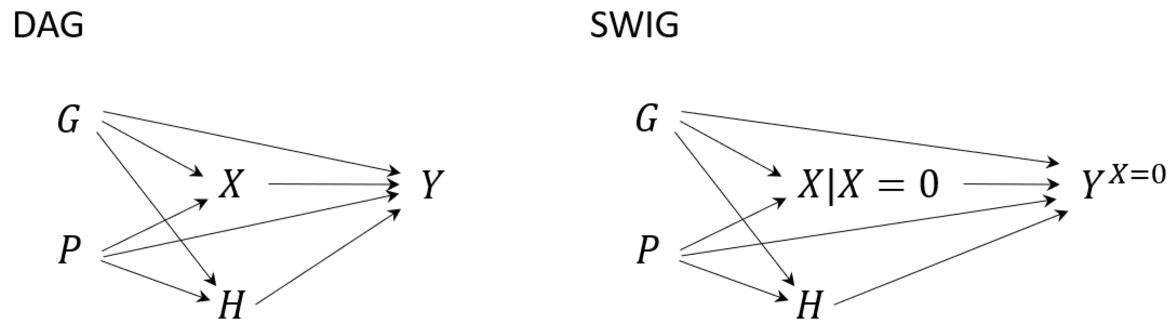

Web Figure 3. DAGs for further scenarios in which the naive treatment effects are biased. U denotes an unmeasured confounder of H and Y. In (a) the adjusted NTE are unbiased. In (b) and (c) the negative control corrected treatment effects (NCCTE) are unbiased under the assumption that there is no product term GP in the model for $Y^{X=0}$, conditional on H and U.

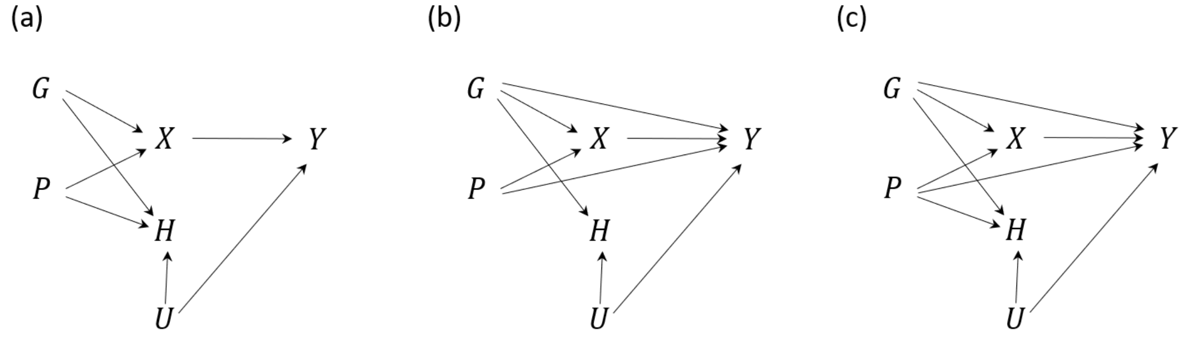

Web Table 1. Estimated naive treatment effect (NTE), negative control effect (NCE) and negative-control-corrected treatment effect (NCCTE) of ivacaftor on **ppFEV₁**, using the time-period comparison (Unadjusted: NTE_P, NCE_P, NCCTE_P. Adjusted: $\mathrm{NTE}_P^{\mathrm{Con}}$, $\mathrm{NCE}_P^{\mathrm{Con}}$, $\mathrm{NCCTE}_P^{\mathrm{Con}}$) and the genotype comparison (Unadjusted: NTE_G, NCE_G, NCCTE_G. Adjusted: $\mathrm{NTE}_G^{\mathrm{Con}}$, $\mathrm{NCE}_G^{\mathrm{Con}}$, $\mathrm{NCCTE}_G^{\mathrm{Con}}$). Including all non-gating mutations in groups C and D. A) Absolute step-change in ppFEV_1 (β_{ST}). B) Absolute change in the annual ppFEV_1 slope (β_{SL}). The adjusted analysis adjusts for baseline variables: sex, age, ethnicity, smoking status, CF-related diabetes, ppFEV_1, IV days (including an indicator of a non-zero count, and a linear term for the non-zero counts), mucolytic treatment use and bacterial infection.

			A) Step-change effect			B) Slope-change effect		
			Est.	95% CI	P	Est.	95% CI	P
Time-period Comparison	Unadjusted	NTE (group B vs A)	5.83	(3.91,7.64)	<0.001	0.41	(-0.67,1.39)	0.430
		NCE (group D vs C)	0.60	(0.12,1.10)	0.019	0.08	(-0.20,0.35)	0.576
		NCCTE	5.23	(3.21,7.09)	<0.001	0.33	(-0.77,1.31)	0.537
	Adjusted	NTE (group B vs A)	7.27	(5.87,8.57)	<0.001	0.68	(0.11,1.32)	0.033
		NCE (group D vs C)	0.77	(0.44,1.08)	<0.001	0.20	(0.04,0.37)	0.020
		NCCTE	6.50	(5.06,7.85)	<0.001	0.49	(-0.15,1.13)	0.146
Genotype Comparison	Unadjusted	NTE (group B vs D)	4.12	(1.40,6.47)	0.001	0.76	(0.10,1.39)	0.018
		NCE (group A vs C)	-1.11	(-3.58,1.27)	0.352	0.42	(-0.37,1.26)	0.310
		NCCTE	5.23	(3.21,7.09)	<0.001	0.33	(-0.77,1.31)	0.537
	Adjusted	NTE (group B vs D)	6.22	(5.17,7.24)	<0.001	0.67	(0.27,1.10)	0.001
		NCE (group A vs C)	-0.37	(-1.36,0.65)	0.462	0.19	(-0.36,0.70)	0.490
		NCCTE	6.59	(5.22,7.90)	<0.001	0.49	(-0.14,1.13)	0.140

Web Table 2. Estimated naive treatment effect (NTE), negative control effect (NCE) and negative-control-corrected treatment effect (NCCTE) of ivacaftor on rate of **IV days**, using the time-period comparison (Unadjusted: NTE_P, NCE_P, NCCTE_P. Adjusted: $\mathrm{NTE}_P^{\mathrm{Con}}$, $\mathrm{NCE}_P^{\mathrm{Con}}$, $\mathrm{NCCTE}_P^{\mathrm{Con}}$) and the genotype comparison (Unadjusted: NTE_G, NCE_G, NCCTE_G. Adjusted: $\mathrm{NTE}_G^{\mathrm{Con}}$, $\mathrm{NCE}_G^{\mathrm{Con}}$, $\mathrm{NCCTE}_G^{\mathrm{Con}}$). Including all non-gating mutations in groups C and D. A) In year 1 (exp(γ_{X1})). B) In year 2 (exp(γ_{X2})). C) In year 3 (exp(γ_{X3})). The adjusted analysis adjusts for baseline variables: sex, age, ethnicity, smoking status, CF-related diabetes, ppFEV_1, IV days (including an indicator of a non-zero count, and a linear term for the non-zero counts), mucolytic treatment use and bacterial infection. RR: rate ratio.

			A) Year 1 effect			B) Year 2 effect			C) Year 3 effect		
			RR	95% CI	P	RR	95% CI	P	RR	95% CI	P
Time-period Comparison	Unadjusted	NTE (group B vs A)	0.50	(0.40,0.61)	<0.001	0.47	(0.36,0.60)	<0.001	0.42	(0.31,0.55)	<0.001
		NCE (group D vs C)	0.93	(0.89,0.97)	0.001	0.89	(0.85,0.93)	<0.001	0.86	(0.81,0.91)	<0.001
		NCCTE	0.54	(0.42,0.66)	<0.001	0.52	(0.41,0.67)	<0.001	0.49	(0.36,0.65)	<0.001
	Adjusted	NTE (group B vs A)	0.42	(0.29,0.54)	<0.001	0.28	(0.21,0.41)	<0.001	0.31	(0.19,0.43)	<0.001
		NCE (group D vs C)	0.77	(0.74,0.83)	<0.001	0.75	(0.70,0.80)	<0.001	0.74	(0.67,0.79)	<0.001
		NCCTE	0.55	(0.36,0.69)	<0.001	0.37	(0.28,0.55)	<0.001	0.42	(0.26,0.58)	<0.001
Genotype Comparison	Unadjusted	NTE (group B vs D)	0.51	(0.40,0.63)	<0.001	0.5	(0.39,0.62)	<0.001	0.44	(0.33,0.54)	<0.001
		NCE (group A vs C)	0.96	(0.80,1.15)	0.611	0.95	(0.81,1.11)	0.485	0.89	(0.72,1.08)	0.252
		NCCTE	0.54	(0.42,0.66)	<0.001	0.52	(0.41,0.67)	<0.001	0.49	(0.36,0.65)	<0.001
	Adjusted	NTE (group B vs D)	0.43	(0.33,0.54)	<0.001	0.38	(0.28,0.53)	<0.001	0.37	(0.27,0.47)	<0.001
		NCE (group A vs C)	0.80	(0.68,0.95)	0.010	0.88	(0.74,1.04)	0.132	0.89	(0.72,1.09)	0.270
		NCCTE	0.53	(0.39,0.70)	<0.001	0.43	(0.31,0.61)	<0.001	0.41	(0.29,0.59)	<0.001

Web Table 3. Estimated naive treatment effect (NTE), negative control effect (NCE) and negative-control-corrected treatment effect (NCCTE) of ivacaftor on **percent predicted forced vital capacity**, using the time-period comparison (Unadjusted: NTE_P, NCE_P, NCCTE_P. Adjusted: $\mathrm{NTE}_P^{\mathrm{Con}}$, $\mathrm{NCE}_P^{\mathrm{Con}}$, $\mathrm{NCCTE}_P^{\mathrm{Con}}$) and the genotype comparison (Unadjusted: NTE_G, NCE_G, NCCTE_G. Adjusted: $\mathrm{NTE}_G^{\mathrm{Con}}$, $\mathrm{NCE}_G^{\mathrm{Con}}$, $\mathrm{NCCTE}_G^{\mathrm{Con}}$). Including all non-gating mutations in groups C and D. A) Absolute step-change in $\mathrm{ppFEV_1}$ (β_{ST}). B) Absolute change in the annual $\mathrm{ppFEV_1}$ slope (β_{SL}). The adjusted analysis adjusts for baseline variables: sex, age, ethnicity, smoking status, CF-related diabetes, percent predicted forced vital capacity, IV days (including an indicator of a non-zero count, and a linear term for the non-zero counts), mucolytic treatment use and bacterial infection.

			A) Step-change effect			B) Slope-change effect		
			Est.	95% CI	P	Est.	95% CI	P
Time-period Comparison	Unadjusted	NTE (group B vs A)	4.76	(3.07,6.38)	<0.001	0.36	(-0.61,1.31)	0.459
		NCE (group D vs C)	0.45	(0.02,0.89)	0.049	-0.02	(-0.26,0.23)	0.888
		NCCTE	4.30	(2.55,6.03)	<0.001	0.38	(-0.61,1.34)	0.450
	Adjusted	NTE (group B vs A)	5.82	(4.50,7.05)	<0.001	0.50	(-0.16,1.18)	0.143
		NCE (group D vs C)	0.51	(0.17,0.85)	0.004	0.03	(-0.15,0.20)	0.770
		NCCTE	5.31	(4.03,6.63)	<0.001	0.48	(-0.24,1.17)	0.182
Genotype Comparison	Unadjusted	NTE (group B vs D)	4.62	(2.56,6.53)	<0.001	0.64	(0.06,1.20)	0.027
		NCE (group A vs C)	0.32	(-1.75,2.22)	0.751	0.26	(-0.47,0.98)	0.485
		NCCTE	4.30	(2.55,6.03)	<0.001	0.38	(-0.61,1.34)	0.450
	Adjusted	NTE (group B vs D)	5.14	(4.13,6.16)	<0.001	0.65	(0.25,1.07)	0.002
		NCE (group A vs C)	-0.26	(-1.22,0.71)	0.609	0.17	(-0.39,0.71)	0.546
		NCCTE	5.39	(4.10,6.72)	<0.001	0.49	(-0.22,1.19)	0.175

Web Table 4. Estimated naive treatment effect (NTE), negative control effect (NCE) and negative-control-corrected treatment effect (NCCTE) of ivacaftor on **percent predicted forced mid-expiratory flow**, using the time-period comparison (Unadjusted: NTE_P, NCE_P, NCCTE_P. Adjusted: $\mathrm{NTE}_P^{\mathrm{Con}}$, $\mathrm{NCE}_P^{\mathrm{Con}}$, $\mathrm{NCCTE}_P^{\mathrm{Con}}$) and the genotype comparison (Unadjusted: NTE_G, NCE_G, NCCTE_G. Adjusted: $\mathrm{NTE}_G^{\mathrm{Con}}$, $\mathrm{NCE}_G^{\mathrm{Con}}$, $\mathrm{NCCTE}_G^{\mathrm{Con}}$). Including all non-gating mutations in groups C and D. A) Absolute step-change in percent predicted forced mid-expiratory flow (β_{ST}). B) Absolute change in the annual percent predicted forced mid-expiratory flow slope (β_{SL}). The adjusted analysis adjusts for baseline variables: sex, age, ethnicity, smoking status, CF-related diabetes, percent predicted forced mid-expiratory flow, IV days (including an indicator of a non-zero count, and a linear term for the non-zero counts), mucolytic treatment use and bacterial infection.

			A) Step-change effect			B) Slope-change effect		
			Est.	95% CI	P	Est.	95% CI	P
Time-period Comparison	Unadjusted	NTE (group B vs A)	6.34	(1.26,11.68)	0.019	1.41	(-2.01,4.34)	0.376
		NCE (group D vs C)	1.04	(-0.24,2.27)	0.102	0.71	(-0.07,1.50)	0.080
		NCCTE	5.30	(-0.06,11.02)	0.056	0.70	(-2.83,3.64)	0.669
	Adjusted	NTE (group B vs A)	7.03	(2.81,11.61)	0.002	1.73	(-0.65,3.86)	0.145
		NCE (group D vs C)	3.43	(2.40,4.40)	<0.001	0.66	(0.08,1.25)	0.029
		NCCTE	3.59	(-0.69,8.29)	0.124	1.06	(-1.45,3.29)	0.385
Genotype Comparison	Unadjusted	NTE (group B vs D)	2.49	(-2.81,8.27)	0.377	1.49	(-0.57,3.26)	0.139
		NCE (group A vs C)	-2.81	(-7.78,2.01)	0.268	0.79	(-1.72,3.49)	0.540
		NCCTE	5.30	(-0.06,11.02)	0.056	0.70	(-2.83,3.64)	0.669
	Adjusted	NTE (group B vs D)	4.98	(2.02,8.15)	0.002	1.14	(-0.27,2.36)	0.086
		NCE (group A vs C)	0.57	(-2.69,3.87)	0.736	0.16	(-1.71,2.18)	0.876
		NCCTE	4.41	(0.15,8.99)	0.046	0.98	(-1.52,3.23)	0.420

Web Table 5. Number of people and total number of longitudinal observations in the UK CF Registry divided into four groups based on genotype (gating ($G = 1$), or other ($G = 0$)) and time-period (pre-ivacaftor (2008-2012) ($P = 0$), or post-ivacaftor (2013-2016) ($P = 1$)). Only including people homozygous or heterozygous for f508del in the 'other' genotype group. Many individuals contribute to both groups A and B or both groups C and D.

Genotype	No. of people	No. of longitudinal observations	No. of people	No. of longitudinal observations
	Group A ($P = 0, G = 1$)		Group B ($P = 1, G = 1$)	
Gating ($G = 1$)	437	1326	397	1368
	Group C ($P = 0, G = 0$)		Group D ($P = 1, G = 0$)	
Other ($G = 0$)	5796	17 472	6675	22 229

Web Table 6. Summary of groups at baseline, defined as 2008 for the pre-ivacaftor period and 2012 for the post-ivacaftor period. Only including those heterozygous or homozygous for f508del in groups C and D.

Variable	Group							
	A $P=0, G=1$ N = 437		B $P=1, G=1$ N = 397		C $P=0, G=0$ N = 5796		D $P=1, G=0$ N = 6675	
	n	%	n	%	n	%	n	%
Ivacaftor Use	0	0	397	100	0	0	0	0
Total Number of Post-baseline Visits[a]	3.0 (1.1)		3.4 (1.1)		3.0 (1.1)		3.3 (1.0)	
Baseline Age (Years)[a]	20.4 (10.8)		22.4 (11.2)		20.6 (11.3)		21.6 (12.3)	
Female	205	46.9	186	46.9	2705	46.7	3138	47.0
White Ethnicity	428	97.9	390	98.2	5683	98.1	6509	97.5
Baseline ppFEV$_1$[a]	71.0 (23.2)		69.7 (23.2)		71.5 (23.3)		71.6 (23.4)	
Baseline Percent Predicted Forced Vital Capacity[a,b]	84.8 (19.4)		84.1 (18.9)		84.1 (19.5)		84.3 (19.6)	
Baseline Percent Predicted Forced Mid-Expiratory Flow[a,b]	56.3 (31.3)		55.9 (32.4)		60.8 (33.0)		58.1 (30.8)	
Baseline IV Days[a]	18.4 (28.1)		20.2 (30.5)		18.0 (28.1)		19.1 (28.8)	
Baseline Infection[c]	358	81.9	350	88.2	4448	76.7	5443	81.5
Baseline CF-Related Diabetes	69	15.8	90	22.7	1118	19.3	1620	24.3
Baseline Smoker	9	2.1	9	2.3	130	2.2	169	2.5
Baseline Mucolytic Treatment[d]	223	51.0	264	66.5	2763	47.7	4428	66.3

[a] Values are expressed as mean (standard deviation).
[b] Percent predicted forced vital capacity based on 13 293 observations, percent predicted forced mid-expiratory flow based on 5223 observations, out of a total of 13 305 individuals across the four groups.
[c] Baseline infection includes *Staphylococcus aureus*, *Pseudomonas aeruginosa*, *Aspergillus fumigatus*, methicillin resistant *Staphylococcus aureus* (MRSA), influenza, *Stenotrophomonas maltophilia* and *Burkholderia cepacia* complex.
[d] Baseline mucolytic treatment includes acetylcysteine, dornase alfa, hypertonic saline and mannitol.

Web Table 7. Estimated naive treatment effect (NTE), negative control effect (NCE) and negative-control-corrected treatment effect (NCCTE) of ivacaftor on **ppFEV$_1$**, using the time-period comparison (Unadjusted: NTE_P, NCE_P, NCCTE_P. Adjusted: $\mathrm{NTE}_P^{\mathrm{Con}}$, $\mathrm{NCE}_P^{\mathrm{Con}}$, $\mathrm{NCCTE}_P^{\mathrm{Con}}$) and the genotype comparison (Unadjusted: NTE_G, NCE_G, NCCTE_G. Adjusted: $\mathrm{NTE}_G^{\mathrm{Con}}$, $\mathrm{NCE}_G^{\mathrm{Con}}$, $\mathrm{NCCTE}_G^{\mathrm{Con}}$). Only including those heterozygous or homozygous for f508del in groups C and D. A) Absolute step-change in ppFEV$_1$ (β_{ST}). B) Absolute change in the annual ppFEV$_1$ slope (β_{SL}). The adjusted analysis adjusts for baseline variables: sex, age, ethnicity, smoking status, CF-related diabetes, ppFEV$_1$, IV days (including an indicator of a non-zero count, and a linear term for the non-zero counts), mucolytic treatment use and bacterial infection.

			A) Step-change effect			B) Slope-change effect		
			Est.	95% CI	P	Est.	95% CI	P
Time-period Comparison	Unadjusted	NTE (group B vs A)	5.83	(4.06,7.64)	<0.001	0.41	(-0.57,1.38)	0.430
		NCE (group D vs C)	0.40	(-0.11,0.94)	0.123	0.10	(-0.17,0.37)	0.486
		NCCTE	5.44	(3.63,7.36)	<0.001	0.31	(-0.72,1.39)	0.568
	Adjusted	NTE (group B vs A)	7.27	(5.95,8.57)	<0.001	0.68	(0.07,1.31)	0.037
		NCE (group D vs C)	0.83	(0.51,1.18)	<0.001	0.19	(0.02,0.37)	0.035
		NCCTE	6.44	(5.09,7.74)	<0.001	0.50	(-0.14,1.19)	0.146
Genotype Comparison	Unadjusted	NTE (group B vs D)	4.49	(1.81,6.93)	0.001	0.70	(0.07,1.34)	0.032
		NCE (group A vs C)	-0.95	(-3.27,1.47)	0.426	0.39	(-0.43,1.20)	0.357
		NCCTE	5.44	(3.63,7.36)	<0.001	0.31	(-0.72,1.39)	0.568
	Adjusted	NTE (group B vs D)	6.22	(5.11,7.40)	<0.001	0.67	(0.30,1.11)	0.001
		NCE (group A vs C)	-0.33	(-1.31,0.61)	0.490	0.18	(-0.35,0.68)	0.503
		NCCTE	6.55	(5.23,7.89)	<0.001	0.50	(-0.14,1.20)	0.147

Web Table 8. Estimated naive treatment effect (NTE), negative control effect (NCE) and negative-control-corrected treatment effect (NCCTE) of ivacaftor on rate of **IV days**, using the time-period comparison (Unadjusted: NTE_P, NCE_P, NCCTE_P. Adjusted: $\mathrm{NTE}_P^{\mathrm{Con}}$, $\mathrm{NCE}_P^{\mathrm{Con}}$, $\mathrm{NCCTE}_P^{\mathrm{Con}}$) and the genotype comparison (Unadjusted: NTE_G, NCE_G, NCCTE_G. Adjusted: $\mathrm{NTE}_G^{\mathrm{Con}}$, $\mathrm{NCE}_G^{\mathrm{Con}}$, $\mathrm{NCCTE}_G^{\mathrm{Con}}$). Only including those heterozygous or homozygous for f508del in groups C and D. A) In year 1 (exp(γ_{X1})). B) In year 2 (exp(γ_{X2})). C) In year 3 (exp(γ_{X3})). The adjusted analysis adjusts for baseline variables: sex, age, ethnicity, smoking status, CF-related diabetes, ppFEV_1, IV days (including an indicator of a non-zero count, and a linear term for the non-zero counts), mucolytic treatment use and bacterial infection. RR: rate ratio.

			A) Year 1 effect			B) Year 2 effect			C) Year 3 effect		
			RR	95% CI	P	RR	95% CI	P	RR	95% CI	P
Time-period Comparison	Unadjusted	NTE (group B vs A)	0.50	(0.37,0.65)	<0.001	0.47	(0.34,0.63)	<0.001	0.42	(0.31,0.57)	<0.001
		NCE (group D vs C)	0.93	(0.88,0.99)	0.019	0.89	(0.84,0.95)	<0.001	0.87	(0.82,0.94)	<0.001
		NCCTE	0.53	(0.40,0.70)	<0.001	0.52	(0.38,0.70)	<0.001	0.48	(0.35,0.66)	<0.001
	Adjusted	NTE (group B vs A)	0.42	(0.30,0.56)	<0.001	0.28	(0.22,0.40)	<0.001	0.31	(0.19,0.42)	<0.001
		NCE (group D vs C)	0.78	(0.75,0.85)	<0.001	0.76	(0.71,0.80)	<0.001	0.76	(0.70,0.81)	<0.001
		NCCTE	0.54	(0.37,0.70)	<0.001	0.36	(0.28,0.53)	<0.001	0.41	(0.26,0.57)	<0.001
Genotype Comparison	Unadjusted	NTE (group B vs D)	0.50	(0.40,0.62)	<0.001	0.49	(0.36,0.61)	<0.001	0.43	(0.33,0.55)	<0.001
		NCE (group A vs C)	0.94	(0.78,1.10)	0.496	0.94	(0.80,1.09)	0.421	0.89	(0.73,1.07)	0.242
		NCCTE	0.53	(0.40,0.70)	<0.001	0.52	(0.38,0.70)	<0.001	0.48	(0.35,0.66)	<0.001
	Adjusted	NTE (group B vs D)	0.42	(0.32,0.53)	<0.001	0.37	(0.27,0.49)	<0.001	0.36	(0.26,0.48)	<0.001
		NCE (group A vs C)	0.80	(0.67,0.92)	0.005	0.87	(0.73,1.03)	0.119	0.89	(0.73,1.09)	0.295
		NCCTE	0.53	(0.39,0.69)	<0.001	0.43	(0.31,0.58)	<0.001	0.40	(0.29,0.57)	<0.001

Web Table 9. Estimated naive treatment effect (NTE), negative control effect (NCE) and negative-control-corrected treatment effect (NCCTE) of ivacaftor on **percent predicted forced vital capacity**, using the time-period comparison (Unadjusted: NTE_P, NCE_P, NCCTE_P. Adjusted: $\mathrm{NTE}_P^{\mathrm{Con}}$, $\mathrm{NCE}_P^{\mathrm{Con}}$, $\mathrm{NCCTE}_P^{\mathrm{Con}}$) and the genotype comparison (Unadjusted: NTE_G, NCE_G, NCCTE_G. Adjusted: $\mathrm{NTE}_G^{\mathrm{Con}}$, $\mathrm{NCE}_G^{\mathrm{Con}}$, $\mathrm{NCCTE}_G^{\mathrm{Con}}$). Only including those heterozygous or homozygous for f508del in groups C and D. A) Absolute step-change in $ppFEV_1$ (β_{ST}). B) Absolute change in the annual $ppFEV_1$ slope (β_{SL}). The adjusted analysis adjusts for baseline variables: sex, age, ethnicity, smoking status, CF-related diabetes, percent predicted forced vital capacity, IV days (including an indicator of a non-zero count, and a linear term for the non-zero counts), mucolytic treatment use and bacterial infection.

			A) Step-change effect			B) Slope-change effect		
			Est.	95% CI	P	Est.	95% CI	P
Time-period Comparison	Unadjusted	NTE (group B vs A)	4.76	(3.12,6.36)	<0.001	0.36	(-0.53,1.31)	0.441
		NCE (group D vs C)	0.28	(-0.18,0.74)	0.225	0.01	(-0.24,0.27)	0.964
		NCCTE	4.47	(2.76,6.16)	<0.001	0.35	(-0.55,1.34)	0.464
	Adjusted	NTE (group B vs A)	5.82	(4.48,7.06)	<0.001	0.50	(-0.16,1.18)	0.131
		NCE (group D vs C)	0.54	(0.16,0.87)	0.004	0.03	(-0.16,0.21)	0.738
		NCCTE	5.28	(3.91,6.66)	<0.001	0.47	(-0.23,1.18)	0.171
Genotype Comparison	Unadjusted	NTE (group B vs D)	4.73	(2.79,6.61)	<0.001	0.62	(0.06,1.16)	0.027
		NCE (group A vs C)	0.25	(-1.48,2.12)	0.789	0.27	(-0.50,1.01)	0.485
		NCCTE	4.47	(2.76,6.16)	<0.001	0.35	(-0.55,1.34)	0.464
	Adjusted	NTE (group B vs D)	5.11	(4.10,6.11)	<0.001	0.66	(0.23,1.10)	0.003
		NCE (group A vs C)	-0.26	(-1.22,0.74)	0.609	0.18	(-0.39,0.71)	0.521
		NCCTE	5.37	(4.03,6.71)	<0.001	0.48	(-0.23,1.18)	0.163

Web Table 10. Estimated naive treatment effect (NTE), negative control effect (NCE) and negative-control-corrected treatment effect (NCCTE) of ivacaftor on **percent predicted forced mid-expiratory flow**, using the time-period comparison (Unadjusted: NTE_P, NCE_P, NCCTE_P. Adjusted: $\mathrm{NTE}_P^{\mathrm{Con}}$, $\mathrm{NCE}_P^{\mathrm{Con}}$, $\mathrm{NCCTE}_P^{\mathrm{Con}}$) and the genotype comparison (Unadjusted: NTE_G, NCE_G, NCCTE_G. Adjusted: $\mathrm{NTE}_G^{\mathrm{Con}}$, $\mathrm{NCE}_G^{\mathrm{Con}}$, $\mathrm{NCCTE}_G^{\mathrm{Con}}$). Only including those heterozygous or homozygous for f508del in groups C and D. A) Absolute step-change in percent predicted forced mid-expiratory flow (β_{ST}). B) Absolute change in the annual percent predicted forced mid-expiratory flow slope (β_{SL}). The adjusted analysis adjusts for baseline variables: sex, age, ethnicity, smoking status, CF-related diabetes, percent predicted forced mid-expiratory flow, IV days (including an indicator of a non-zero count, and a linear term for the non-zero counts), mucolytic treatment use and bacterial infection.

			A) Step-change effect			B) Slope-change effect		
			Est.	95% CI	P	Est.	95% CI	P
Time-period Comparison	Unadjusted	NTE (group B vs A)	6.34	(0.98,11.59)	0.019	1.41	(-1.81,4.52)	0.378
		NCE (group D vs C)	0.79	(-0.50,2.07)	0.233	0.70	(-0.05,1.46)	0.077
		NCCTE	5.55	(0.45,10.84)	0.046	0.71	(-2.68,3.91)	0.667
	Adjusted	NTE (group B vs A)	7.03	(2.61,11.51)	0.003	1.73	(-0.59,4.00)	0.148
		NCE (group D vs C)	3.35	(2.26,4.31)	<0.001	0.62	(0.03,1.27)	0.046
		NCCTE	3.67	(-0.64,8.45)	0.128	1.10	(-1.29,3.46)	0.373
Genotype Comparison	Unadjusted	NTE (group B vs D)	2.81	(-2.40,8.29)	0.315	1.46	(-0.43,3.32)	0.131
		NCE (group A vs C)	-2.73	(-7.65,2.04)	0.267	0.75	(-1.70,3.43)	0.562
		NCCTE	5.55	(0.45,10.84)	0.046	0.71	(-2.68,3.91)	0.667
	Adjusted	NTE (group B vs D)	4.99	(1.95,8.08)	0.001	1.16	(-0.05,2.50)	0.078
		NCE (group A vs C)	0.50	(-3.05,3.66)	0.769	0.16	(-1.78,2.12)	0.871
		NCCTE	4.49	(0.40,9.03)	0.051	1.00	(-1.45,3.47)	0.412

Web Table 11. Number of people and total number of longitudinal observations in the UK CF Registry divided into four groups based on genotype (gating ($G = 1$), or other ($G = 0$)) and time-period (pre-ivacaftor (2008-2012) ($P = 0$), or post-ivacaftor (2013-2016) ($P = 1$)). Only including people homozygous for f508del in the 'other' genotype group. Many individuals contribute to both groups A and B or both groups C and D.

Genotype	No. of people	No. of longitudinal observations	No. of people	No. of longitudinal observations
	Group A ($P = 0, G = 1$)		Group B ($P = 1, G = 1$)	
Gating ($G = 1$)	437	1326	397	1368
	Group C ($P = 0, G = 0$)		Group D ($P = 1, G = 0$)	
Other ($G = 0$)	3488	10 636	3934	13 282

Web Table 12. Summary of groups at baseline, defined as 2008 for the pre-ivacaftor period and 2012 for the post-ivacaftor period. Only including those homozygous for f508del in groups C and D.

Variable	Group							
	A $P=0, G=1$ N = 437		B $P=1, G=1$ N = 397		C $P=0, G=0$ N = 3488		D $P=1, G=0$ N = 3934	
	n	%	n	%	n	%	n	%
Ivacaftor Use	0	0	397	100	0	0	0	0
Total Number of Post-baseline Visits[a]	3.0 (1.1)		3.4 (1.1)		3.0 (1.1)		3.4 (1.0)	
Baseline Age (Years)[a]	20.4 (10.8)		22.4 (11.2)		19.2 (9.8)		20.0 (10.6)	
Female	205	46.9	186	46.9	1571	45.0	1797	45.7
White Ethnicity	428	97.9	390	98.2	3433	98.4	3853	97.9
Baseline ppFEV$_1$[a]	71.0 (23.2)		69.7 (23.2)		70.8 (23.0)		70.5 (23.3)	
Baseline Percent Predicted Forced Vital Capacity[a,b]	84.8 (19.4)		84.1 (18.9)		83.8 (19.5)		83.6 (19.6)	
Baseline Percent Predicted Forced Mid-Expiratory Flow[a,b]	56.3 (31.3)		55.9 (32.4)		59.4 (32.8)		56.4 (30.5)	
Baseline IV Days[a]	18.4 (28.1)		20.2 (30.5)		19.1 (28.5)		20.8 (28.8)	
Baseline Infection[c]	358	81.9	350	88.2	2724	78.1	3319	84.4
Baseline CF-Related Diabetes	69	15.8	90	22.7	756	21.7	1078	27.4
Baseline Smoker	9	2.1	9	2.3	75	2.2	91	2.3
Baseline Mucolytic Treatment[d]	223	51.0	264	66.5	1762	50.5	2776	70.6

[a] Values are expressed as mean (standard deviation).
[b] Percent predicted forced vital capacity based on 8254 observations, percent predicted forced mid-expiratory flow based on 3328 observations, out of a total of 8256 individuals across the four groups.
[c] Baseline infection includes *Staphylococcus aureus*, *Pseudomonas aeruginosa*, *Aspergillus fumigatus*, methicillin resistant *Staphylococcus aureus* (MRSA), influenza, *Stenotrophomonas maltophilia* and *Burkholderia cepacia* complex.
[d] Baseline mucolytic treatment includes acetylcysteine, dornase alfa, hypertonic saline and mannitol.

Web Table 13. Estimated naive treatment effect (NTE), negative control effect (NCE) and negative-control-corrected treatment effect (NCCTE) of ivacaftor on **ppFEV₁**, using the time-period comparison (Unadjusted: NTE_P, NCE_P, NCCTE_P. Adjusted: $\mathrm{NTE}_P^{\mathrm{Con}}$, $\mathrm{NCE}_P^{\mathrm{Con}}$, $\mathrm{NCCTE}_P^{\mathrm{Con}}$) and the genotype comparison (Unadjusted: NTE_G, NCE_G, NCCTE_G. Adjusted: $\mathrm{NTE}_G^{\mathrm{Con}}$, $\mathrm{NCE}_G^{\mathrm{Con}}$, $\mathrm{NCCTE}_G^{\mathrm{Con}}$). Only including those homozygous for f508del in groups C and D. A) Absolute step-change in $ppFEV_1$ (β_{ST}). B) Absolute change in the annual $ppFEV_1$ slope (β_{SL}). The adjusted analysis adjusts for baseline variables: sex, age, ethnicity, smoking status, CF-related diabetes, $ppFEV_1$, IV days (including an indicator of a non-zero count, and a linear term for the non-zero counts), mucolytic treatment use and bacterial infection.

			A) Step-change effect			B) Slope-change effect		
			Est.	95% CI	P	Est.	95% CI	P
Time-period Comparison	Unadjusted	NTE (group B vs A)	5.83	(4.13,7.59)	<0.001	0.41	(-0.61,1.47)	0.446
		NCE (group D vs C)	0.23	(-0.34,0.93)	0.482	0.09	(-0.25,0.46)	0.605
		NCCTE	5.60	(3.68,7.46)	<0.001	0.32	(-0.72,1.43)	0.574
	Adjusted	NTE (group B vs A)	7.27	(5.92,8.67)	<0.001	0.68	(0.08,1.30)	0.035
		NCE (group D vs C)	1.08	(0.62,1.55)	<0.001	0.15	(-0.09,0.38)	0.210
		NCCTE	6.19	(4.72,7.65)	<0.001	0.53	(-0.10,1.17)	0.117
Genotype Comparison	Unadjusted	NTE (group B vs D)	5.55	(2.98,7.83)	<0.001	0.73	(0.09,1.42)	0.033
		NCE (group A vs C)	-0.05	(-2.39,2.28)	0.963	0.42	(-0.49,1.22)	0.333
		NCCTE	5.60	(3.68,7.46)	<0.001	0.32	(-0.72,1.43)	0.574
	Adjusted	NTE (group B vs D)	6.37	(5.28,7.49)	<0.001	0.79	(0.36,1.26)	<0.001
		NCE (group A vs C)	-0.05	(-1.10,0.91)	0.919	0.28	(-0.30,0.82)	0.313
		NCCTE	6.42	(5.00,7.85)	<0.001	0.51	(-0.15,1.15)	0.134

Web Table 14. Estimated naive treatment effect (NTE), negative control effect (NCE) and negative-control-corrected treatment effect (NCCTE) of ivacaftor on rate of **IV days**, using the time-period comparison (Unadjusted: NTE_P, NCE_P, NCCTE_P. Adjusted: $\mathrm{NTE}_P^{\mathrm{Con}}$, $\mathrm{NCE}_P^{\mathrm{Con}}$, $\mathrm{NCCTE}_P^{\mathrm{Con}}$) and the genotype comparison (Unadjusted: NTE_G, NCE_G, NCCTE_G. Adjusted: $\mathrm{NTE}_G^{\mathrm{Con}}$, $\mathrm{NCE}_G^{\mathrm{Con}}$, $\mathrm{NCCTE}_G^{\mathrm{Con}}$). Only including those homozygous for f508del in groups C and D. A) In year 1 (exp(γ_{X1})). B) In year 2 (exp(γ_{X2})). C) In year 3 (exp(γ_{X3})). The adjusted analysis adjusts for baseline variables: sex, age, ethnicity, smoking status, CF-related diabetes, ppFEV_1, IV days (including an indicator of a non-zero count, and a linear term for the non-zero counts), mucolytic treatment use and bacterial infection. RR: rate ratio.

			A) Year 1 effect			B) Year 2 effect			C) Year 3 effect		
			RR	95% CI	P	RR	95% CI	P	RR	95% CI	P
Time-period Comparison	Unadjusted	NTE (group B vs A)	0.50	(0.39,0.61)	<0.001	0.47	(0.36,0.59)	<0.001	0.42	(0.31,0.56)	<0.001
		NCE (group D vs C)	0.96	(0.91,1.01)	0.117	0.94	(0.89,1.00)	0.048	0.91	(0.85,0.98)	0.017
		NCCTE	0.52	(0.40,0.65)	<0.001	0.49	(0.39,0.62)	<0.001	0.46	(0.34,0.62)	<0.001
	Adjusted	NTE (group B vs A)	0.42	(0.29,0.56)	<0.001	0.28	(0.21,0.42)	<0.001	0.31	(0.19,0.41)	<0.001
		NCE (group D vs C)	0.82	(0.77,0.88)	<0.001	0.79	(0.72,0.84)	<0.001	0.79	(0.73,0.87)	<0.001
		NCCTE	0.51	(0.34,0.67)	<0.001	0.35	(0.27,0.53)	<0.001	0.39	(0.24,0.52)	<0.001
Genotype Comparison	Unadjusted	NTE (group B vs D)	0.46	(0.36,0.57)	<0.001	0.44	(0.34,0.56)	<0.001	0.39	(0.30,0.49)	<0.001
		NCE (group A vs C)	0.88	(0.74,1.05)	0.184	0.89	(0.76,1.04)	0.173	0.85	(0.68,1.03)	0.137
		NCCTE	0.52	(0.40,0.65)	<0.001	0.49	(0.39,0.62)	<0.001	0.46	(0.34,0.62)	<0.001
	Adjusted	NTE (group B vs D)	0.40	(0.30,0.50)	<0.001	0.36	(0.26,0.47)	<0.001	0.34	(0.25,0.44)	<0.001
		NCE (group A vs C)	0.77	(0.64,0.90)	0.002	0.84	(0.69,0.99)	0.047	0.87	(0.71,1.06)	0.165
		NCCTE	0.52	(0.38,0.69)	<0.001	0.43	(0.30,0.58)	<0.001	0.39	(0.28,0.54)	<0.001

Web Table 15. Estimated naive treatment effect (NTE), negative control effect (NCE) and negative-control-corrected treatment effect (NCCTE) of ivacaftor on **percent predicted forced vital capacity**, using the time-period comparison (Unadjusted: NTE_P, NCE_P, NCCTE_P. Adjusted: $\mathrm{NTE}_P^{\mathrm{Con}}$, $\mathrm{NCE}_P^{\mathrm{Con}}$, $\mathrm{NCCTE}_P^{\mathrm{Con}}$) and the genotype comparison (Unadjusted: NTE_G, NCE_G, NCCTE_G. Adjusted: $\mathrm{NTE}_G^{\mathrm{Con}}$, $\mathrm{NCE}_G^{\mathrm{Con}}$, $\mathrm{NCCTE}_G^{\mathrm{Con}}$). Only including those homozygous for f508del in groups C and D. A) Absolute step-change in ppFEV_1 (β_{ST}). B) Absolute change in the annual ppFEV_1 slope (β_{SL}). The adjusted analysis adjusts for baseline variables: sex, age, ethnicity, smoking status, CF-related diabetes, percent predicted forced vital capacity, IV days (including an indicator of a non-zero count, and a linear term for the non-zero counts), mucolytic treatment use and bacterial infection.

			A) Step-change effect			B) Slope-change effect		
			Est.	95% CI	P	Est.	95% CI	P
Time-period Comparison	Unadjusted	NTE (group B vs A)	4.76	(3.08,6.31)	<0.001	0.36	(-0.46,1.27)	0.431
		NCE (group D vs C)	0.20	(-0.38,0.81)	0.522	0.00	(-0.34,0.29)	0.982
		NCCTE	4.56	(2.82,6.21)	<0.001	0.36	(-0.50,1.31)	0.447
	Adjusted	NTE (group B vs A)	5.82	(4.51,7.02)	<0.001	0.50	(-0.17,1.20)	0.152
		NCE (group D vs C)	0.84	(0.36,1.29)	0.001	0.04	(-0.21,0.27)	0.763
		NCCTE	4.98	(3.59,6.30)	<0.001	0.47	(-0.24,1.21)	0.205
Genotype Comparison	Unadjusted	NTE (group B vs D)	5.29	(3.22,7.31)	<0.001	0.67	(0.10,1.25)	0.025
		NCE (group A vs C)	0.73	(-1.26,2.70)	0.458	0.30	(-0.42,1.04)	0.407
		NCCTE	4.56	(2.82,6.21)	<0.001	0.36	(-0.50,1.31)	0.447
	Adjusted	NTE (group B vs D)	5.08	(4.00,6.03)	<0.001	0.73	(0.30,1.19)	0.001
		NCE (group A vs C)	-0.04	(-1.06,0.95)	0.941	0.28	(-0.30,0.83)	0.330
		NCCTE	5.11	(3.73,6.41)	<0.001	0.46	(-0.24,1.16)	0.216

Web Table 16. Estimated naive treatment effect (NTE), negative control effect (NCE) and negative-control-corrected treatment effect (NCCTE) of ivacaftor on **percent predicted forced mid-expiratory flow**, using the time-period comparison (Unadjusted: NTE_P, NCE_P, NCCTE_P. Adjusted: $\mathrm{NTE}_P^{\mathrm{Con}}$, $\mathrm{NCE}_P^{\mathrm{Con}}$, $\mathrm{NCCTE}_P^{\mathrm{Con}}$) and the genotype comparison (Unadjusted: NTE_G, NCE_G, NCCTE_G. Adjusted: $\mathrm{NTE}_G^{\mathrm{Con}}$, $\mathrm{NCE}_G^{\mathrm{Con}}$, $\mathrm{NCCTE}_G^{\mathrm{Con}}$). Only including those homozygous for f508del in groups C and D. A) Absolute step-change in percent predicted forced mid-expiratory flow (β_{ST}). B) Absolute change in the annual percent predicted forced mid-expiratory flow slope (β_{SL}). The adjusted analysis adjusts for baseline variables: sex, age, ethnicity, smoking status, CF-related diabetes, percent predicted forced mid-expiratory flow, IV days (including an indicator of a non-zero count, and a linear term for the non-zero counts), mucolytic treatment use and bacterial infection.

			A) Step-change effect			B) Slope-change effect		
			Est.	95% CI	P	Est.	95% CI	P
Time-period Comparison	Unadjusted	NTE (group B vs A)	6.34	(1.32,11.87)	0.021	1.41	(-1.78,4.48)	0.391
		NCE (group D vs C)	1.09	(-0.55,2.79)	0.204	0.58	(-0.49,1.60)	0.275
		NCCTE	5.25	(-0.07,11.07)	0.069	0.83	(-2.49,4.22)	0.629
	Adjusted	NTE (group B vs A)	7.03	(2.08,11.49)	0.003	1.73	(-0.89,3.98)	0.152
		NCE (group D vs C)	3.60	(2.31,4.90)	<0.001	0.18	(-0.67,0.99)	0.648
		NCCTE	3.43	(-1.77,8.10)	0.159	1.54	(-1.06,3.87)	0.213
Genotype Comparison	Unadjusted	NTE (group B vs D)	4.28	(-1.08,9.51)	0.122	1.35	(-0.65,3.21)	0.168
		NCE (group A vs C)	-0.97	(-5.91,3.63)	0.701	0.53	(-1.98,3.07)	0.683
		NCCTE	5.25	(-0.07,11.07)	0.069	0.83	(-2.49,4.22)	0.629
	Adjusted	NTE (group B vs D)	5.57	(2.30,8.83)	0.001	1.20	(-0.15,2.56)	0.079
		NCE (group A vs C)	1.52	(-1.69,5.26)	0.375	-0.12	(-2.16,1.95)	0.903
		NCCTE	4.05	(-0.69,8.30)	0.077	1.32	(-1.06,3.68)	0.281
